# Salivary IgA and vimentin differentiate *in vitro* SARS-CoV-2 infection: A study of 290 convalescent COVID-19 patients

**DOI:** 10.1016/j.mucimm.2023.11.007

**Published:** 2024-02

**Authors:** Samuel Ellis, Rosie Way, Miranda Nel, Alice Burleigh, Ivan Doykov, Japhette Kembou-Ringert, Maximillian Woodall, Tereza Masonou, Katie-Marie Case, Arturo Torres Ortez, Timothy D. McHugh, Antonio Casal, Laura E. McCoy, Sudaxshina Murdan, Robert E. Hynds, Kimberly C. Gilmour, Louis Grandjean, Mario Cortina-Borja, Wendy E Heywood, Kevin Mills, Claire M. Smith

**Affiliations:** 1 UCL Great Ormond Street Institute of Child Health, London, UK; 2 Centre for Adolescent Rheumatology, University College London, London, UK; 3 UCL Centre for Clinical Microbiology, Royal Free Hospital, London, UK; 4 Institute of Immunity and Transplantation, Division of Infection and Immunity, University College London, London, UK; 5 Department of Pharmaceutics, UCL School of Pharmacy, London, UK; 6 Epithelial Cell Biology in ENT Research (EpiCENTR) Group, Developmental Biology and Cancer Department, UCL Great Ormond Street Institute of Child Health, London, UK; 7 Great Ormond Street Hospital NHS Foundation Trust, London, UK

## Abstract

SARS-CoV-2 initially infects cells in the nasopharynx and oral cavity. The immune system at these mucosal sites plays a crucial role in minimizing viral transmission and infection. To develop new strategies for preventing SARS-CoV-2 infection, this study aimed to identify proteins that protect against viral infection in saliva.

We collected 551 saliva samples from 290 healthcare workers who had tested positive for COVID-19, before vaccination, between June and December 2020. The samples were categorized based on their ability to block or enhance infection using *in vitro* assays. Mass spectrometry and enzyme-linked immunosorbent assay experiments were used to identify and measure the abundance of proteins that specifically bind to SARS-CoV-2 antigens.

Immunoglobulin (Ig)A specific to SARS-CoV-2 antigens was detectable in over 83% of the convalescent saliva samples. We found that concentrations of anti-receptor-binding domain IgA >500 pg/µg total protein in saliva correlate with reduced viral infectivity *in vitro*. However, there is a dissociation between the salivary IgA response to SARS-CoV-2, and systemic IgG titers in convalescent COVID-19 patients. Then, using an innovative technique known as spike-baited mass spectrometry, we identified novel spike-binding proteins in saliva, most notably vimentin, which correlated with increased viral infectivity *in vitro* and could serve as a therapeutic target against COVID-19.

## INTRODUCTION

SARS-CoV-2 infections continue to cause substantial morbidity and mortality worldwide. Despite efforts to develop effective treatments, few options are currently available for infected individuals, while vaccination remains the most effective preventive strategy. Therefore, identifying novel correlates for protection against SARS-CoV-2 infection is crucial for the development of new preventive and therapeutic strategies for COVID-19.

The initial target of the SARS-CoV-2 virus is the epithelial cells lining the mucosal surface of the nasal passages, nasopharynx, and oral cavity, where it binds to the host angiotensin-converting enzyme 2 (ACE2) receptor via its spike protein, thereby initiating infection^1^. Blocking this interaction at the mucosal site is crucial for preventing infection. Secretory immunoglobulin (Ig)A is the primary antibody mediator in mucosal secretions and has been used as a reliable biomarker for mucosal antibody responses to other respiratory viruses, such as influenza^2^, ^3^. Antibodies against the viral spike protein, particularly against the spike receptor-binding domain (RBD), can effectively inhibit viral entry into cells^4^. While initial studies have mainly focused on the seroconversion of IgG and IgM as useful metrics for population analysis^5^, ^6^, ^7^, ^8^, mucosal immunity is increasingly being recognized as a crucial factor for protection^9^, ^10^. As the primary sites of viral challenge in the nasopharynx, mucosal factors, including immunoglobulins and other secreted proteins, have the potential to neutralize the virus before infection can be established^4^, ^11^.

**Fig. 1:**
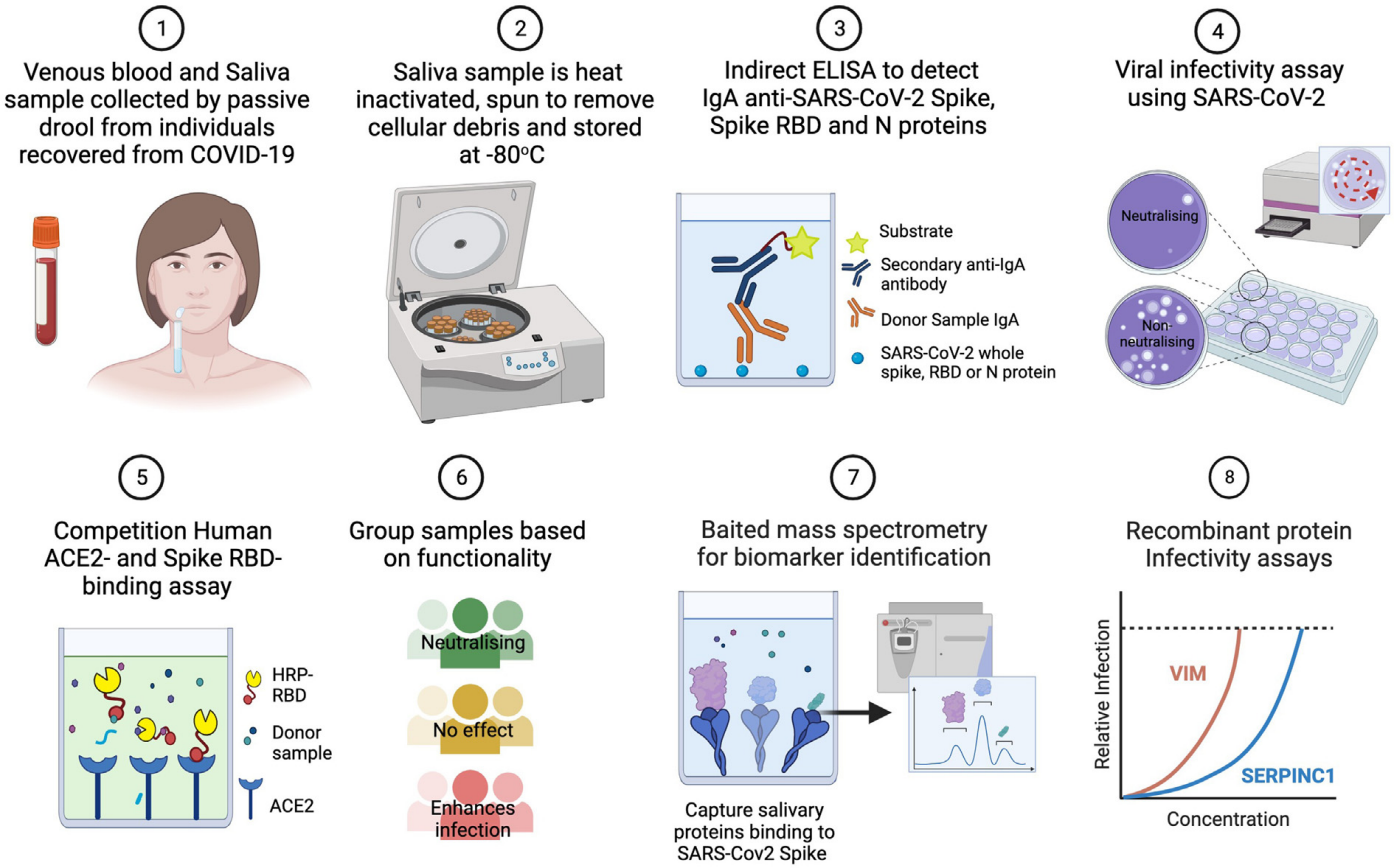
Schematic of methods used to study mucosal correlates of protection against *in vitro* SARS-CoV-2 infection in saliva. Created with Biorender.com. ACE2 = angiotensin-converting enzyme 2.

In this study, we analyzed saliva collected from a cohort of 290 pre-vaccinated healthcare workers at London’s Great Ormond Street Hospital for Children who had tested positive for COVID-19. Saliva offers numerous advantages for screening, including its high accessibility and non-invasive collection, which is particularly important when considering future applications of the approach in a wider population^12^. Saliva also serves as an abundant surrogate sample for nasal secretions, particularly in the absence of rhinorrhea^13^. Saliva is known to contain immune effectors that are present in the nasopharynx, including systemically derived IgG and antimicrobial proteins such as mucins, lactoferrin, lysozyme, peroxidases, and defensins^14^, ^15^. Here, we employed a functional and proteomic approach to determine the most abundant salivary proteins that correlate with the neutralization of SARS-CoV-2 infection (Fig. 1).

## RESULTS

### Participant characteristics

The study collected 551 saliva samples from 290 healthcare workers (HCWs) who worked at London’s Great Ormond Street Hospital (GOSH) over 5 months before the COVID-19 vaccine was rolled out (June–December 2020). All participants had tested positive for COVID-19 during the first UK wave, as confirmed by positive polymerase chain reaction (PCR) or serological screening through a staff testing program at GOSH. Participants were invited to provide samples at monthly follow-up clinic visits, with each participant providing between one and five samples for longitudinal analysis (Supplementary Fig. 1A). The study included active hospital staff aged between 19 and 66 years old, with a mean age of 38 years (Supplementary Fig. 1B). Around 75.2% of participants identified as female, and 72.8% were of White ethnicity (Supplementary Figs. 1B and 1C). The majority of participants (around 70%) were nurses or allied health professionals (Supplementary Fig. 1D). This study population is broadly representative of the documented workforce demographics at GOSH^16^, particularly matching the greater proportion of female staff at 75.5%.

During the first wave of infections (March–May 2020) in the UK, the symptoms of COVID-19 were primarily defined as a high temperature and a new, continuous cough. Although other indicative symptoms such as anosmia were common, many cases were asymptomatic^17^. In our cohort, around 60.7% (*n* = 176) of convalescent participants self-reported having experienced symptoms associated with COVID-19 (Supplementary Fig. 1E), including anosmia (44.1%), cough (46.6%), and fever (37.2%) (Supplementary Table 1). In total, 39.3% of participants described themselves as asymptomatic, despite testing positive for SARS-CoV-2 through PCR and/or serology screening, and only 12% of participants reported having relevant underlying medical conditions (Supplementary Fig. 1F).

This participant population also provided matched serum samples as part of the Co-Stars study^18^. We were, therefore, able to analyze the serum IgG status of these individuals at the matched donation times for their saliva samples. We found that 85% of participants were concurrently serologically positive for anti-SARS-CoV-2 IgG (Supplementary Fig. 1G).

### Salivary anti-RBD IgA response to SARS-CoV-2 correlates with viral neutralization

**Fig. 2:**
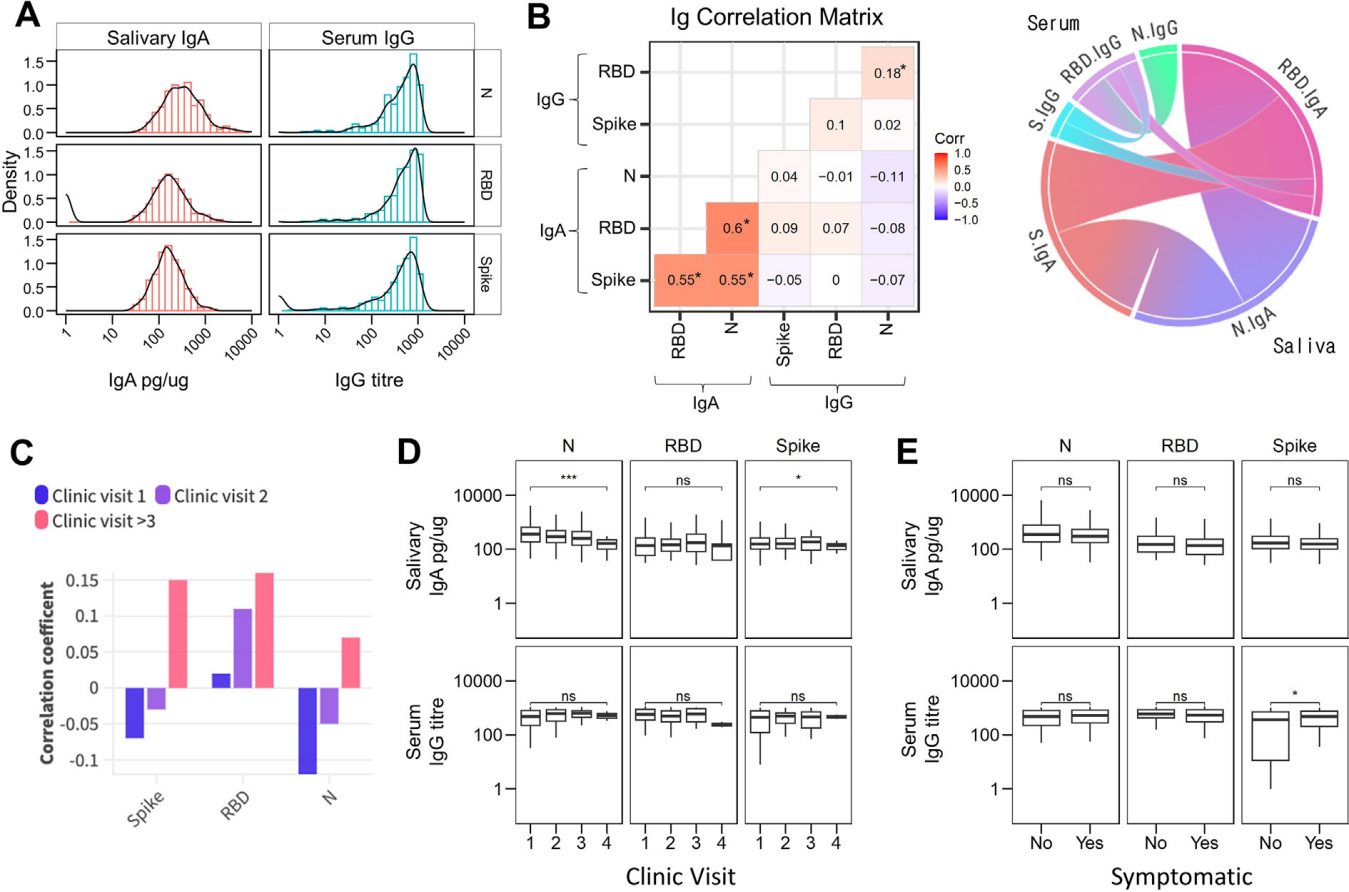
Analysis of antibodies against Spike, RBD and Nucleocapsid antigens in health care workers after recovery from COVID-19 in absence of prior vaccination. (A) Distribution of antibody responses to SARS-CoV-2 spike (S), RBD and nucleocapsid antigens for salivary IgA (n = 488) and serum IgG (n = 172). IgA is displayed as pg/μg total protein as measured by ELISA, IgG is displayed as MSD chemiluminescent assay titer. (B) Left panel shows the correlation matrix showing the association of anti-SARS-CoV-2 saliva IgA and systemic IgG by antigen across all timepoints as determined using the cor function of ggcorrplot package using R version 2023.03.1+446. Right panel shows a chord diagram of the results of the correlation matrix. Data excludes Ab responses <1, n = 172 donors. * represents significant correlation *p* < 0.05. C, Correlation of serum IgG and salivary IgA across different clinic visits. D, Salivary IgA and serum IgG responses in longitudinal samples at sequential clinic visits. E, Distribution of salivary IgA and serum IgG against SARS-CoV-2 antigens for self-reported symptomatic versus asymptomatic participants. Ab = antibody; ELISA = enzyme-linked immunosorbent assay; Ig = immunoglobulin; MSD = Meso Scale Discovery; RBD = receptor binding domain.

To investigate the mucosal immune response, we first measured secretory IgA, a crucial immunological factor. We analyzed a total of 488 saliva samples for IgA recognizing three SARS-CoV-2 antigens: trimeric spike (S), nucleocapsid (N), and spike RBD using enzyme-linked immunosorbent assay (ELISA). We found that 86% (422 out of 488) of the samples were positive for IgA antibodies binding to the SARS-CoV-2 S protein, of these 85% (418 out of 488) were also positive for SARS-CoV-2 N protein, and 83% (377 out of 450) were positive for SARS-CoV-2 spike RBD. To ensure consistency across samples with highly variable viscosities, IgA concentrations were normalized to total protein (pg/µg) for further analysis (Fig. 2A). Samples containing detectable IgA against multiple SARS-CoV-2 proteins showed strong correlations (Fig. 2B, Supplementary Fig. 2A). We then compared salivary IgA and serum IgG for all samples with matched saliva and serum samples. Here we found an absence of correlation (*R*^2^ = −0.11 − 0.09, *p* > 0.05) between the saliva IgA and serum IgG titers (Fig. 2B, Supplementary Fig. 2B). While serum and saliva antibody titers did not significantly correlate for any antigen, we did observe a trend for an increased correlation coefficient at later sampling times (Fig. 2C).

Out of the 290 convalescent participants, 113 provided >1 sample at intervals of approximately 3–5 weeks (Supplementary Table 2). These samples were used to investigate the persistence of salivary IgA against SARS-CoV-2 antigens. The stability of IgA concentration over time was analyzed using a constant exponential decay model for all three antigens (Supplementary Fig. 2C). The slope of this model (Supplementary Fig. 2D) was used to calculate a decay rate of −0.94% per day [95% confidence interval (CI) = −1.33 to −0.54%] for anti-N IgA. In contrast, anti-S and anti-RBD IgA levels remained stable at 0.04% (95% CI = −0.51 to 0.60%) and 0.48% (95% CI = −0.14 to 1.11%), respectively. IgA concentrations significantly decreased by the fourth sample for antigens S and N but not RBD when examining repeated samples per individual donor yet did not significantly decline in the concurrent matched IgG titers (Fig. 2D).

Self-reported COVID-19 symptoms were only associated with a higher IgG titer against spike antigen (Fig. 2E). When analyzing any effect of ethnicity, black participants exhibited lower IgA responses to the S and N antigens compared to other participants (Supplementary Fig. 2E). Anti-N antigen IgA levels showed a significant decrease beyond 60 years of age (Supplementary Fig. 2F). To assess the potential cross-reactivity of IgA antibodies with coronavirus antigens, we conducted ELISA testing on a subset of saliva samples (n = 14). These samples were selected to represent the spectrum of anti-SARS-CoV-2 IgA titers detected. Specifically, we examined their reactivity against the S and N proteins derived from two seasonal coronaviruses, OC43 and 229E. We found that the anti-S IgA titers remained consistent across the strains, while the anti-N IgA response was significantly (*p* < 0.05) lower against OC43 and 229E antigens compared to SARS-CoV-2 (Supplementary Fig. 2G).

### Functional analysis of saliva samples: protein concentration, pH, epithelial cell toxicity, in vitro infectivity, and RBD:ACE2 inhibition

We then investigated the impact of convalescent saliva using *in vitro* SARS-CoV-2 infection and other functional antiviral assays. Saliva samples can have different densities and alkalinity levels, which could interfere with viral cell infection. To account for this, we first measured their total protein concentration and pH. We found that our saliva samples had a median protein concentration of 779 µg/ml (interquartile range of 542–1137 µg/ml, *n* = 489) (Supplementary Fig. 3A) and a median pH of 7.37 (interquartile range of 6.88–7.86, *n* = 48) (Supplementary Fig. 3B).

### *In vitro* infectivity

**Fig. 3:**
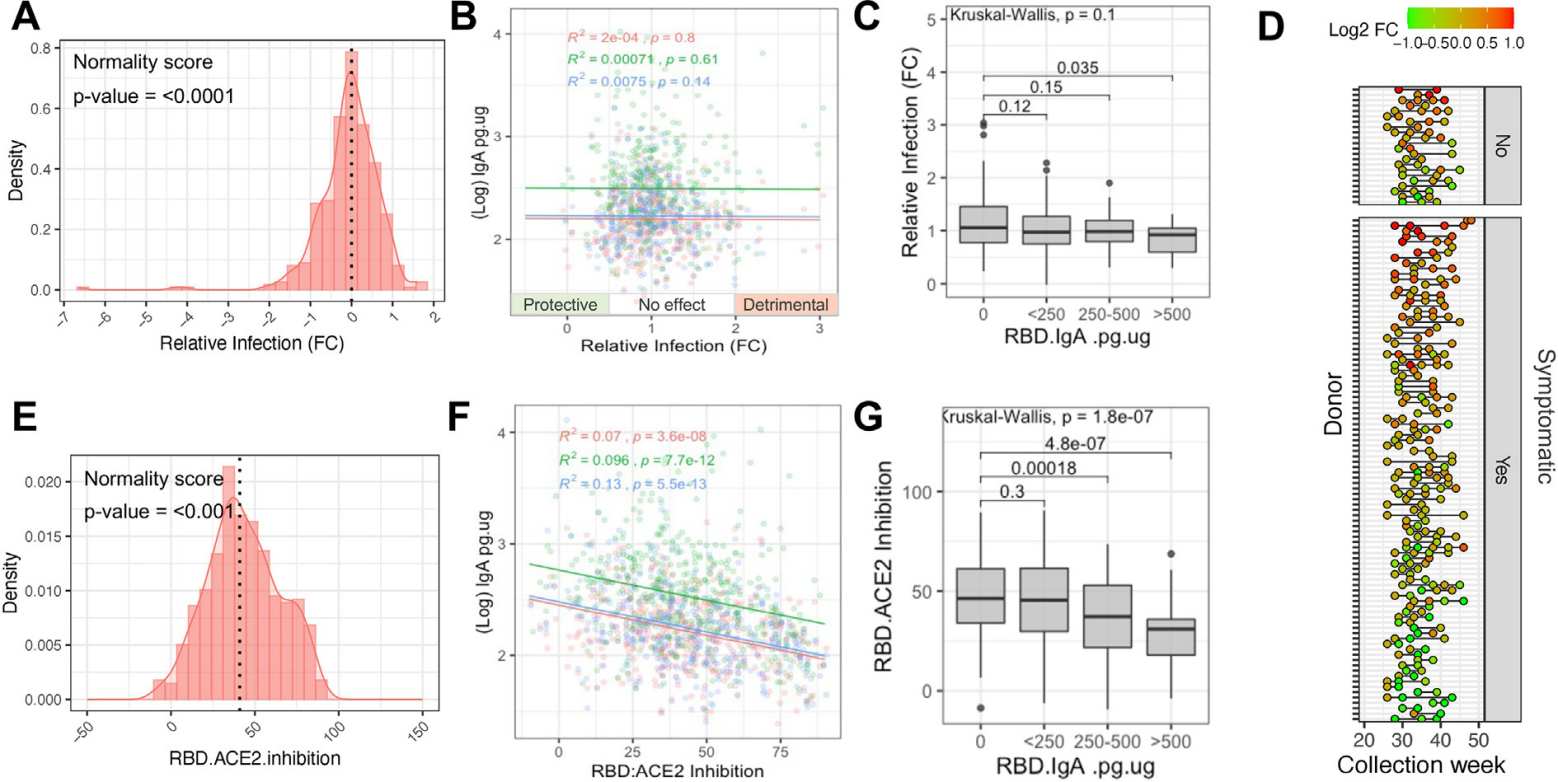
Viral neutralization and RBD-ACE2 inhibition by saliva from health care workers after recovery from confirmed COVID-19 in absence of prior vaccination. (A) Distribution of relative infection of VeroE6 cells (fold-change versus virus-only controls) after pre-incubation of virus with saliva (n = 395). Shapiro-Wilk test of normality, where *p* value < 0.05 indicates significant deviation from a normal distribution. (B) The relationship between IgA level and relative SARS-CoV-2 infection of VeroE6 cells for S (red), RBD (blue) and N (green). (C) Relative infection analyzed against anti-RBD IgA titer, with solid black bars denoting the median per group. (D) Longitudinal reproducibility of neutralization for donors who provided multiple samples, ordered by average log2 fold change in infectivity per donor, and separated into symptomatic and non-symptomatic groups. (E) Distribution of saliva ability to inhibit the interaction of SARS-CoV-2 RBD with human ACE2 receptor, relative to positive and negative controls (n = 490). (F) A linear model fit was used to show the relationship between saliva IgA and inhibition of in vitro RBD-ACE2 binding for antigens S (red), RBD (blue) and N (green). (G) Inhibition of RBD-ACE2 binding analyzed against anti-RBD IgA titer, with solid black bars denoting the median per group. ACE2 = angiotensin-converting enzyme 2; RBD = receptor binding domain.

We found that saliva alone was not cytotoxic to uninfected VeroE6 cells, while pre-incubation with saliva significantly reduced (*p* < 0.001) the amount of VeroE6 cell death caused by the SARS-CoV-2 infection (Supplementary Figs. 3C and 3D). Our findings revealed a weak positive correlation (*R*^2^ = 0.013, *p* = 0.027) between higher salivary protein content and reduced infectivity of VeroE6 cells (Supplementary Fig. 3E). Most saliva samples had minimal impact on infectivity, with an overall mean log2 fold-change in infectivity of –0.08 (Fig. 3A). However, a proportion of samples (*n* = 29 samples, from 26 distinct donors, 7.3%) exhibited a greater than 2-fold reduction in infectivity, while another subset (*n* = 13 samples, from 11 distinct donors, 3.3%) showed a greater than 2-fold increase in SARS-CoV-2 infectivity, despite not having impacted cell viability in the absence of virus (these samples will subsequently be referred to as 'detrimental' samples).

The effect on SARS-CoV-2 infectivity did not correlate with IgA titers at the population level (Fig. 3B). However, samples with the highest concentrations of anti-RBD IgA in saliva (>500 pg/µg total protein) significantly (*p* = 0.035) reduced viral infection compared to samples negative for anti-RBD IgA (Fig. 3C). We found no significant association between the effect of saliva on VeroE6 cell infectivity, and the anti-SARS-CoV-2 IgG density in matched serum samples (Supplementary Fig. 3F).

Participants who provided multiple sample donations, showed a trend for consistently protective or detrimental samples (Fig. 3D). This was consistent for donors with and without symptoms and suggests the importance of intrinsic and conserved donor characteristics rather than transient sampling variables.

### RBD:ACE2 binding inhibition

We found a significant association (*R*^2^ = 0.053, *p* < 0.01) between higher total protein content and the inhibition of RBD-ACE2 binding in competitive ELISA (Supplementary Fig. 3G). Overall, 37.3% of the samples demonstrated a reduction in RBD-ACE2 binding by over 50%, with a median reduction of 41.9% (n = 491) (Fig. 3E). Unexpectedly, the RBD-ACE2 competition ELISA did not show any enhanced inhibition with higher IgA levels and showed an overall negative correlation, which may be partly driven by the normalization of the ELISA quantification against total protein (Fig. 3F). An anti-RBD IgA concentration above 250 pg/µg was associated with significantly less inhibition of RBD-ACE2 binding (Fig. 3G). The magnitude of RBD-ACE2 inhibition caused by saliva also did not correlate with the matched serum IgG data from the donors (Supplementary Fig. 3H).

### Proteomics reveals novel salivary proteins associated with reduction or enhancement of in vitro SARS-CoV-2 infection

**Fig. 4:**
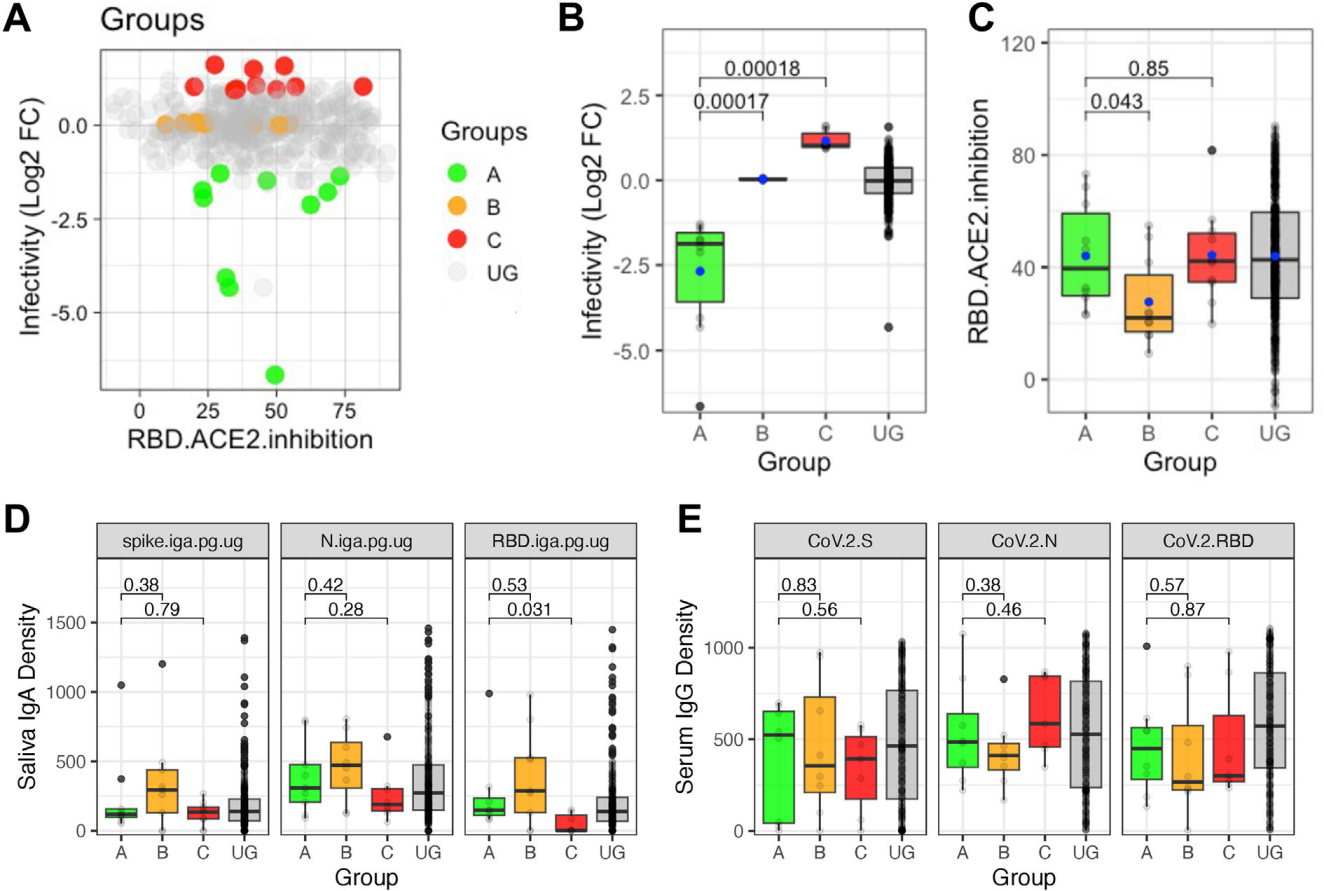
Functional saliva subsets by effect on SARS-CoV-2 infectivity. (A) A graphical representation of sample collection broken down by function. Protective effect on infectivity represents FC >+0.5, detrimental effect represents FC <−0.5. RBD-ACE2 inhibition represents >50% relative inhibition. (B) The relative VeroE6 infection data for each of the samples in subgroups A, B, and C selected for proteomic analysis (n = 10 per group), compared to all other UG samples (n = 365). (C) The relative RBD-ACE2 inhibition data for each of the samples in subgroups A, B, and C selected for proteomic analysis (n = 10 per group), compared to all other UG samples (n = 365). (D) The distribution of salivary IgA concentrations (pg/μg total protein) for the saliva samples in each subgroup, compared to all other UG samples. (E) The distribution of matched serum IgG titers (MSD units) for the saliva samples in each subgroup, compared to UG samples. ACE2 = angiotensin-converting enzyme 2; MSD = Meso Scale Discovery; RBD = receptor binding domain; UG = ungrouped.

To identify novel salivary proteins associated with the *in vitro* viral infectivity, a subset of samples (n = 30) was selected for analysis by mass spectrometry (Supplementary Fig. 4A). Based on their relative infectivity, the samples were divided into three groups: Group A (top 5% neutralizing samples with median fold-change of infectivity = −1.89), Group B (control samples with median fold-change of infectivity = +0.03), and Group C (detrimental samples with the top 5% highest infection with median fold-change of infectivity = +1.02) (Figs. 4A and 4B). Group A exhibited significantly higher inhibition of RBD-ACE2 binding compared to Group B (*p* = 0.043), but no significant difference was found compared to Group C (Fig. 4C). Total protein concentration and pH did not differ significantly among these subgroups or compared to the full ungrouped (UG) sample collection (Supplementary Figs. 4B and 4C). Corresponding to the previous overall findings (Fig. 3C), the detrimental Group C samples had significantly lower anti-RBD IgA, but the subgroups otherwise had similar IgA levels (Fig. 4D). Similarly, the functional subgroups also did not show significant differences in sample-matched serology IgG titers (Fig. 4E). Participant metadata and neutralization data for individual samples in these functional groups are summarized in Supplementary Table 3.

**Fig. 5:**
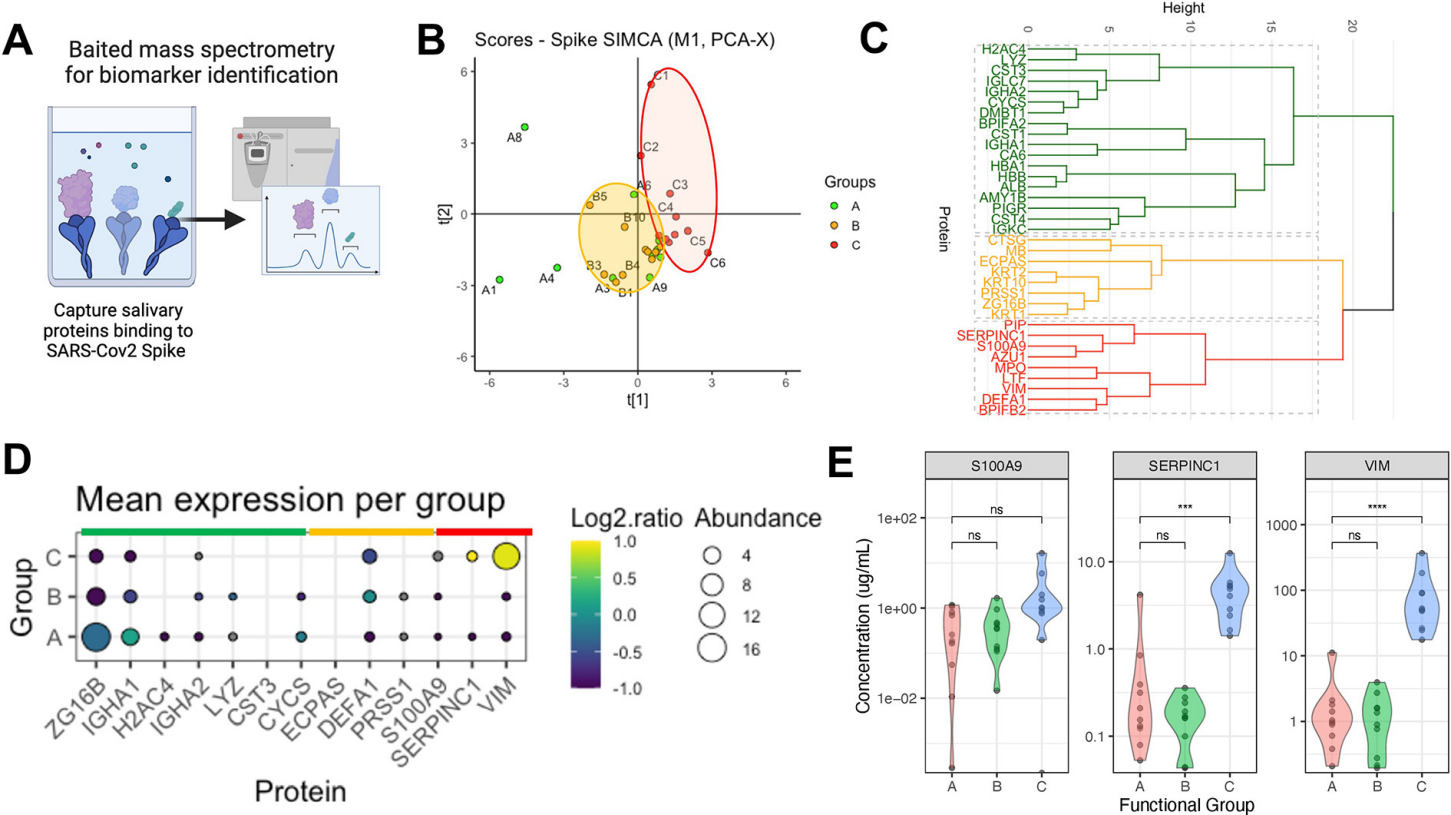
Proteins associated with SARS-CoV-2 infectivity of saliva functional subgroups. (A) Graphical representation of SARS-CoV-2 spike-baited mass spectrometry methodology. Created with Biorender.com. (B) Orthogonal Projections to Latent Structures Discriminant Analysis (OPLS-DA) models between functional groups showing the score plot of the salivary proteins that bind to SARS-CoV-2 spike. (C) Cluster dendrogram of all spike binding proteins detected in saliva. (D) Dot plot showing comparative mean abundance of significantly elevated proteins in groups A–C. (E) Violin plot showing the abundance of most differentially expressed spike-binding proteins detected in Group C (detrimental). Estimated concentration (μg/ml) calculated from mass spectrometry abundance normalized against known mass of spike bait protein (n = 10).

Mass spectrometry and proteomic analysis of these samples were then conducted to examine differences in salivary proteins among the functional groups. A pull-down step with a fixed mass of spike protein was used to first enrich the samples for components with specific affinity for SARS-CoV-2 antigen (Fig. 5A). The relative abundance of all salivary proteins identified from the baited assay is provided in Supplementary Table 4. Orthogonal Projections to Latent Structures Discriminant Analysis (OPLS-DA) models were created to compare group pairs: A versus B, A versus C, and B versus C. While Group A samples appeared to separate from Group B on the OPLS-DA score plot, the separation between A and B was not significant (CV-analysis of variance analysis), suggesting minimal proteome profile differences between the two groups (Fig. 5B). However, Group A was significantly different from Group C by OPLS-DA (*p* = 0.0399), indicating distinct proteome profiles between these groups (Fig. 5B).

Univariable analyses revealed 14 proteins that were elevated in Group A compared to Group C and five proteins that were elevated in Group C compared to Group A (Supplementary Fig. 5A). These proteins formed three clusters based on their relative abundance and function (Fig. 5C). Gene set enrichment analysis identified principal pathways associated with the elevated proteins in each group. Group A proteins were associated with homeostasis and antimicrobial defense pathways, while Group C proteins were associated with pathogen defense and neutrophil degranulation pathways (Supplementary Figs. 5B and 5C).

The most abundant and discriminatory proteins in Group A were IGHA1, ZG16B, IGHA2, and H2AC4 (Fig. 5D), although only IGHA1 and ZG16B were significantly higher than groups B and C (Supplementary Fig. 5D). An association of increased IgA in Group A was indicated by the greater abundance of specific constant heavy chain peptides (IGHA1 and IGHA2), and immunoglobulin constant light chain components [Ig lambda constant 7 (IGLC7) and IGKC] which may correspond to IgA or other immunoglobulins.

For Group C, the prominent proteins identified were vimentin (VIM), S100 calcium-binding protein A9 (S100A9), and antithrombin III (SERPINC1) (Fig. 5D and Supplementary Fig. 5E). By normalizing the mass spectrometry data against the known starting mass of spike protein used as bait, we estimated the relative protein abundance in the saliva samples. Both SERPINC1 and VIM were significantly enhanced in Group C, with VIM the most discriminatory target protein (Fig. 5E). S100A9 positively correlated with VIM and SERPINC1 (Supplementary Fig. 5F). We confirmed that the abundance of VIM was not significantly associated with participant age for this set of samples (Supplementary Fig. 5G). To validate these findings in a different assay we completed preliminary analysis of a subset of saliva by ELISA. Here we found a slightly higher mean VIM concentration in Group C samples compared to non-Group C samples (*p* = 0.09) (Supplementary Fig. 5H).

### Concentrations of vimentin present in saliva enhance in vitro SARS-CoV-2 infection

**Fig. 6:**
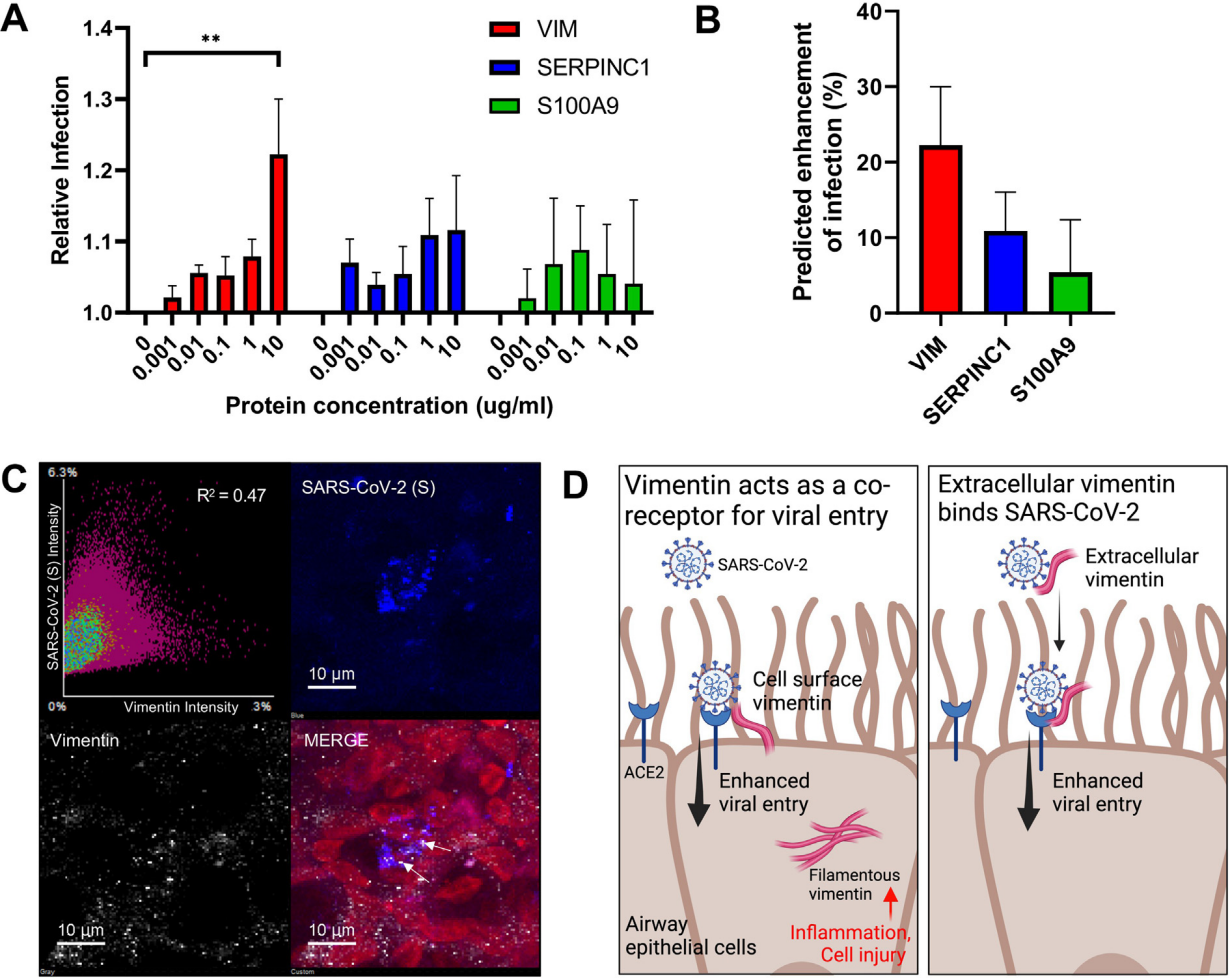
Vimentin concentration in detrimental saliva is associated with enhanced in vitro SARS-CoV-2 infectivity. (A) The effect of increasing concentrations of recombinant proteins on relative SARS-CoV-2 infection of VeroE6 cells (n = 3; ** = *p* < 0.005, analysis of variance). (B) Predicted effect of target proteins on relative infection of VeroE6 cells for vimentin abundances in Group C (detrimental) saliva samples as calculated from mass spectrometry (n = 10). (C) Immunofluorescence staining of infected air-liquid interface airway epithelial cells, with cell nuclei (red), spike antigen (blue) and vimentin (white), and a plot of signal co-localization between SARS-CoV-2 spike and cellular vimentin. (D) Graphical representation of proposed mechanisms for enhanced viral entry caused by vimentin. Left panel shows that surface-expressed vimentin may act as a co-receptor for SARS-CoV-2 and ACE2 binding to promote viral entry. Right panel shows extracellular vimentin in mucosal secretions such as saliva may bind to SARS-CoV-2 via the spike protein, stabilizing binding interactions with ACE2 during cell infection. Created with Biorender.com. ACE2 = angiotensin-converting enzyme 2.

To confirm whether the “detrimental” proteins enhanced SARS-CoV-2 infection, we conducted *in vitro* VeroE6 infection assays using purified recombinant VIM, SERPINC1, and S100A9 across the range of concentrations detected in the saliva. The results showed that a concentration of 10 μg/mL of VIM significantly increased SARS-CoV-2 infection (*p* = 0.005) (Fig. 6A). Although trends toward increased infection were also observed for SERPINC1 and S100A9, these did not reach statistical significance. We further estimated the relative enhancement of VeroE6 infection that would be predicted for each target protein alone, based on the individual salivary concentrations previously detected by proteomics in the Group C samples (Fig. 6B).

Given previous studies indicating that VIM can facilitate viral entry and replication^19^, ^20^, we investigated whether vimentin is associated with SARS-CoV-2 infected nasal epithelial cells. We found that after 72 hours post-infection, SARS-CoV-2 spike protein and VIM co-localized in SARS-CoV-2 infected primary human differentiated nasal epithelial cells grown at the air-liquid interface, with an *R*^2^ value of 0.47 (Fig. 6C). This suggests a possible interaction between these two proteins during SARS-CoV-2 infection in the initially targeted nasal epithelial cells. Based on the findings of this study, as well as other previously reported associations of vimentin with viral cell uptake discussed in detail below, we propose that extracellular vimentin also acts as a co-receptor to enhance SARS-CoV-2 viral entry (Fig. 6D).

## DISCUSSION

In this study, saliva samples collected from HCWs following recovery from COVID-19 were screened to investigate their effect on *in vitro* SARS-CoV-2 infection. We found subsets of saliva samples associated with increased or reduced infection of VeroE6 cells, which is a well-characterized cell line known for its susceptibility to the early-lineage SARS-CoV-2 virus^21^, ^22^. The effect of saliva on infectivity was not attributable to pH or total protein density, and we observed a trend where participants consistently exhibited either neutralizing or detrimental saliva across multiple monthly donations. This supports our initial hypothesis that donor-specific differences in mucosal proteins, rather than any transient properties of the saliva at the point of collection, influenced SARS-CoV-2 infectivity.

### Anti-SARS-CoV-2 IgA is detectable and persistent in saliva, but only high titer anti-RBD IgA is associated with in vitro neutralization

Using our in-house ELISA we detected anti-SARS-CoV-2 IgA in saliva samples at rates of 86.5% (S), 95.7% (N), and 83.8% (spike RBD). Notably, the N antigen showed the highest rate of positive IgA testing, consistent with previous literature^23^, ^24^. Other studies differ in detection rates, likely due to variations in saliva collection methods and IgA measurement techniques. For example, Alkharaan et al. reported a 55.6% positivity rate for IgA recognition of spike in saliva collected within the first 3 months of convalescence^25^, while another study found that IgA to spike or nucleocapsid antigens could be detected in 92.1% of saliva samples within 4 months post-symptom onset^26^. In our study, we were able to detect anti-SARS-CoV-2 IgA in saliva for more than 6 months post-symptom onset. Of the three antigens we tested, only anti-N IgA showed a significant decline over time, whereas anti-S and anti-RBD IgA levels remained relatively stable. These findings align with a study by Fröberg et al.^11^, which demonstrated elevated mucosal IgA against the S and RBD antigens for at least 9 months after mild COVID-19. Interestingly, a previous analysis of serum IgG responses in our HCW cohort showed stable anti-S IgG levels at 200 days post-symptom onset, while anti-N antibodies also declined more rapidly^18^. Some vaccine studies have also reported persistent salivary IgA titers following natural infection, often at higher levels than in vaccinated individuals^27^, ^28^.

Although our data on salivary IgA persistence showed a similar trend to previously published serum IgG modelling^18^, we found no significant correlation between the magnitude of IgA and IgG responses from matched saliva and serum samples. While some studies have reported good correlation between antibodies in these two sample types^29^, ^30^, others have found a poor correlation between salivary IgA and serum IgG^31^. For SARS-CoV-2, anti-S IgA in mucosal fluids has been reported in the absence of seropositivity^32^. Any dissociation is important to the understanding of mucosal protection following COVID-19 vaccine seroconversion, particularly as specific mucosal secretory IgA induction by mRNA vaccination is minimal without pre-exposure to SARS-CoV-2^33^. Confounding factors may also include cross-reactivity of secretory IgA in saliva from other seasonal coronavirus exposures^34^. Our findings suggest that measuring saliva IgA may serve as an effective indicator of prior infection and mucosal response, but it may not reliably reflect systemic immune status.

Regarding donor demographics, individuals aged 60 years and older had lower anti-N IgA titers. This is consistent with literature demonstrating decreased SARS-CoV-2-specific IgA levels with age in nasal fluids of naturally infected patients, and data from vaccination response studies^32^, ^35^, ^36^. Lower overall production of IgA in mucosal secretions has long been associated with immunosenescence^37^, yet the findings of our study only show a significant decrease for anti-N IgA rather than a general decline for all antigens. When comparing by ethnicity, White participants showed higher IgA compared to Asian (anti-N) and Black (anti-N and anti-S) donors. Some serological studies have identified higher anti-SARS-CoV-2 IgG antibody levels in non-White ethnic groups^38^, ^39^, ^40^, and ethnicity has been linked to relative risks of SARS-CoV-2 infection and severe disease^41^, yet there is limited previous research on ethnicity differences in mucosal IgA response to COVID-19.

We found that samples with anti-RBD IgA greater than 500 pg/µg total protein significantly reduced SARS-CoV-2 infection in our VeroE6 cell model. This is the first report, to our knowledge, of a cell protective threshold of anti-SARS-CoV-2 IgA in saliva *in vitro*. Previous studies that examined the antiviral properties of saliva using ELISA-based IgA measurements used arbitrary unit scales^42^, ^43^, making direct comparison to neutralizing antibody concentrations difficult, but have reported an association between anti-RBD IgA levels and *in vitro* neutralization activity. Unexpectedly, we found no positive correlation between anti-RBD IgA concentration and direct inhibition of RBD-ACE2 binding using a competitive ELISA assay, with observed inhibition associated with greater total protein content. This suggests the range of neutralization activity caused by saliva in our VeroE6 model is not driven only by anti-SARS-CoV-2 IgA blocking ACE2 binding.

To elucidate the salivary proteins associated with *in vitro* SARS-CoV-2 infectivity, we utilized a spike-baited mass spectrometry technique. This focused the proteomic investigation on factors with a specific affinity to SARS-CoV-2 spike antigen, which supports a potential effect on viral entry and has previously been used for sensitive analysis of immunoprotein responses in human blood and saliva^44^. The detection of IGHA1 associated with neutralizing saliva provides additional support to our previous findings from direct ELISA assays. Additionally, we identified several other spike-binding IgA protein components, including IgA heavy chain (IGHA2) and light chain Ig lambda constant 7 (IGLC7), as well as the polymeric immunoglobulin receptor. These findings align with the well-established role of IgA as the primary secretory immunoglobulin involved in mucosal innate immunity^9^. Of the other proteins associated with reduced infectivity, lysozyme is a powerful antimicrobial enzyme that exhibits both antibacterial and antiviral properties, including against SARS-CoV-2^45^. Zymogen Granule Protein 16B (ZG16B) is a relatively unexplored secretory lectin protein that is abundantly expressed in human salivary gland tissue^46^. Although an association with SARS-CoV-2 has not been previously reported, ZG16B has been linked to anti-influenza activity in saliva and may interfere with viral interaction with cell-surface receptors through its carbohydrate-binding properties^47^. The diverse range of other identified protein factors aligns with the concept of salivary immune defense networks, where multiple secreted proteins and immunoglobulins work together synergistically to provide antimicrobial activity^14^, ^48^.

### Vimentin is the most discriminatory salivary protein associated with enhanced in vitro SARS-CoV-2 infection

Our proteomics analysis also identified proteins associated with increased susceptibility to SARS-CoV-2 infection *in vitro*, including vimentin (VIM), S100A9 (a component of the calprotectin dimer), and antithrombin III (SERPINC1). Antithrombin III has previously been linked to protection against SARS-CoV-2 by inhibiting the serine protease TMPRSS2^49^, therefore a direct connection to the observed detrimental effect in this model is unclear. Calprotectin, an alarmin involved in immune system activation, has been proposed as a potential biomarker for severe COVID-19 due to its role in cytokine storm development^50^. The presence of S100A9 suggests neutrophil activation and respiratory tissue stress, which may indicate the presence of other pro-inflammatory and stress response factors contributing to the increased sensitivity of VeroE6 cells to viral cytotoxicity^51^.

However, the most discriminatory protein in the detrimental saliva Group was vimentin, and we have further demonstrated that recombinant vimentin alone enhanced *in vitro* infectivity in our epithelial cell infection model. While vimentin is primarily known as an intracellular cytoskeletal protein, it can also be found in extracellular secreted and cell-surface forms^52^. Vimentin expression has been associated with inflammation, cell injury, and epithelial-mesenchymal transition^53^. Interestingly, it can also act as a receptor for various bacterial and viral pathogens, including SARS-CoV^54^, ^55^, ^56^. Our microscopy observations showed co-localization of vimentin and SARS-CoV-2 at infected cells using a human nasal epithelial model. Our results, using a more biologically representative primary cell model, are supported by similar reports of vimentin-spike interactions previously observed in epithelial cell lines^19^, ^53^.

Vimentin has been proposed as a possible binding factor or co-receptor for the SARS-CoV-2 spike protein, with specific interactions between extracellular vimentin and S1 RBD^57^. Studies using epithelial cell models have shown vimentin enhances SARS-CoV-2 spike-pseudotyped virus entry, but importantly the effect was entirely ACE2 dependant^19^, ^20^. As well as cell-surface expression, purified extracellular vimentin also increases pseudotyped virus entry to ACE2/HEK-293 or ACE2/A549 cells^20^. Studies have further demonstrated that the addition of antibodies to block vimentin leads to a reduction in spike-mediated *in vitro* infectivity^19^, ^58^, with others proposing that vimentin could be a future target for SARS-CoV-2 therapeutic interventions^54^, ^58^, ^59^, ^60^. Vimentin in saliva has been investigated as a biomarker for oral cancers, but not to our knowledge in relation to viral infection susceptibility^61^. Additionally, while our study is focused on extracellular vimentin and initial viral entry, others have described how dynamic rearrangement of intracellular vimentin within infected cells can further facilitate viral replication, assembly, and egress^62^.

While further research is needed to fully elucidate the mechanisms involved, our findings, in the context of the literature, support a co-receptor model whereby vimentin in extracellular mucosa and at the cell surface promotes stronger binding of SARS-CoV-2 spike protein to primary receptors such as ACE2, enhancing viral entry (Fig. 6D). Higher levels of vimentin in saliva could thus enhance viral uptake and contribute to the increased infection observed within our detrimental saliva subset. Therefore, high levels of vimentin detectable in saliva may serve as a significant indicator of enhanced mucosal susceptibility to SARS-CoV-2. To our knowledge, our study is the first to demonstrate that measurably raised salivary vimentin in human mucosal samples is associated with increased *in vitro* SARS-CoV-2 infectivity.

### Study limitations

One limitation of our study is the inherent variability of saliva, which can be influenced by external factors such as the time of day and an individual's hydration and consumption. To mitigate this, we implemented strict guidelines for sample acquisition, including collecting samples in a fixed clinic time window and imposing criteria such as no eating, drinking, or smoking in the 30 minutes prior to collection. We employed the passive drool collection method, considered the gold standard for saliva collection, as it avoids active stimulation and provides reliable immunoglobulin sensitivity compared to cotton-based absorption devices^63^, ^64^. We also normalized saliva IgA concentrations against total salivary protein, a common approach in salivary immunoglobulin studie^65^, ^66^.

Vimentin emerged as a promising therapeutic target in this work with enhanced abundance in saliva samples causing increased *in vitro* infectivity. However, there was only scope in this study to test vimentin levels in a subset of samples by mass spectrometry and ELISA. We acknowledge that for future analysis of vimentin as a therapeutic or biomarker target in SARS-CoV-2 susceptibility, it is crucial to conduct broader mucosal vimentin level screening in larger and diverse cohorts.

We utilized a previously described SARS-CoV-2 spike-baited mass spectrometry protocol^44^, which allowed for a focused analysis of salivary protein targets with a specific affinity for the viral antigen. However, we acknowledge that this approach limits the interpretation of other factors in saliva that may indirectly influence infection in our *in vitro* models.

Another limitation of our study was the lack of pre-pandemic saliva samples from our participant population. Therefore, we were unable to thoroughly assess the contribution of pre-existing cross-reactive IgA from previous human coronavirus infections, although we did demonstrate IgA recognition was not higher for antigens from seasonal coronaviruses OC43 and 229E versus SARS-CoV-2. Comparing such pre-pandemic samples would be valuable, as there is increasing evidence of antibody recognition of SARS-CoV-2 antigens in non-infected populations, including IgA responses in saliva^34^, ^67^, ^68^. Understanding the presence and influence of pre-existing cross-reactive antibodies could have significant implications for infection risk and disease severity, especially in non-vaccinated populations.

## CONCLUSION

We found that concentrations of anti-RBD IgA >500 pg/µg total protein in saliva correlate with reduced viral infectivity *in vitro*. However, there is a dissociation between the salivary IgA response to SARS-CoV-2, and systemic IgG titers in convalescent COVID-19 patients. We have also identified spike-binding proteins in saliva, most notably vimentin, which correlates with increased viral infectivity *in vitro* and could serve as a therapeutic target for COVID-19.

## METHODS

### Participants and research ethics approval

Participants were recruited from GOSH NHS Foundation Trust as part of the Health Research Authority approved project Co-Stars (IRAS 282713) between 22nd June 2020 and 23rd November 2020, and prior to vaccine rollout. All participants provided informed written consent. Inclusion criteria included: HCW ≥18 years of age who tested positive for SARS-CoV-2 as part of the staff testing program, with confirmed detectable serological antibodies to SARS-CoV-2 infection and/or a prior positive SARS-CoV-2 PCR result. Exclusion criteria included: <18 years of age, on immunosuppressive or immunomodulatory medication, have received any blood product including immunoglobulins after September 2019, has received convalescent sera as treatment, current diagnosis of a malignancy that may impact test reliability, or those lacking capacity to provide informed consent. Eligible participants were asked to complete a standardized self-reporting questionnaire to provide information on age, medical conditions, occupation, ethnic background (BAME), number of children, home occupancy, and symptoms.

### Sample collection

Participants were asked not to eat, drink, or smoke at least 30 minutes prior to collection. Subsequently, saliva samples were collected by passive drool into a 15ml Falcon tube. Participants were requested to produce minimum of 2 ml sample or maximum produced in 10 minutes. Samples were centrifuged (1000*g*, 5′) to remove cellular debris and heated to 56 °C for 30 minutes to inactivate any infectious virus before aliquoting and storage at −80 °C. Samples were excluded by visible blood or foreign material contamination, or if too mucoid to obtain sufficient supernatant, resulting in 488 individual samples passing quality control for subsequent assays. As part of the Co-Stars project, matching serum samples were collected at the same time as the saliva, which was analyzed independently of this study^18^. Titers of serum IgG were obtained using the Meso Scale Discovery (MSD) Chemiluminescent assay that simultaneously detects and quantifies anti-SARS-CoV-2 IgG specific for trimeric S protein, RBD, and N^18^.

### Total protein quantification

All saliva samples were analyzed for total protein content as measured using PierceTM BCA Protein Assay Kit (23227, Pierce, Waltham, MA, USA), measured on a FLUOstar Optima microplate reader (BMG Labtech, Ottenberg, Germany) at absorbance 584nm.

### Indirect ELISAs for detecting salivary IgA against SARS-CoV-2

In-house indirect ELISAs were developed for the determination of IgA antibodies to recombinant coronavirus antigens: re-fusion trimeric spike glycoprotein (S), nucleocapsid (N) (S and N antigens from SARS-CoV-2, OC43 and 229E were kindly provided by Svend Kjaer and Peter Cherepanov at The Francis Crick Institute, UK) and SARS-CoV-2 spike receptor-binding domain (RBD) (Sinobiological Eschborn, Germany 40592-VNAH-SIB). We used 96-well ELISA plates (Nunc Maxisorp) coated with 50 µL antigen solution at 2 µg/ml in carbonate coating buffer (pH 9.6), overnight at 4 °C. A standard curve (10–160 ng/ml) of human IgA isotype control was also adsorbed (31148, Invitrogen, Waltham, MA, USA). Wells were blocked with 2% BSA in PBS (P4417, Sigma-Aldrich, Gillingham, Dorset, UK), for 1 hour at RT. Samples were diluted 1:4 and 1:16 in PBS-T assay diluent (0.1% Tween-20) and incubated in wells for 1 hour RT. Wells were washed thrice with PBS-T before the addition of horseradish peroxidase (HRP)-conjugated goat anti-human IgA antibody (5104-2404, Bio-Rad, Hercules, CA, USA) at 1:2500 dilution in PBS-T, for 1 hour at RT. Wells were washed as before and developed using TMB High Sensitivity Solution (421501, Biolegend, San Diego, CA, USA) for 10 minutes at RT. 0.2M sulphuric acid was used as a stop solution for the reaction. A FLUOstar Optima microplate reader (BMG Labtech, Ottenberg, Germany) was used to measure optical density at an absorbance of 450 nm.

A subset of saliva samples was analyzed using the Human Vimentin ELISA Kit (ab246526, Abcam, Cambridge, UK), following the supplied protocol.

### Modeling of IgA persistence

A constant exponential decay model was applied to the longitudinal IgA sample data to calculate the decay rate. This was performed as previously described in detail for the serology arm of the Co-Stars study^18^.

### Competitive ELISA for inhibition of RBD-ACE2 binding assay

The ability of proteins in the saliva samples to block the interaction of spike RBD and immobilized Human ACE2 protein was tested by a modified protocol using an anti-SARS-CoV-2 Neutralizing Antibody Titer Serologic Assay Kit (ACROBiosystems, Newark, DE, USA). Here, salivary samples (and positive and negative controls from the kit) were mixed 1:1 with HRP-conjugated SARS-CoV-2 spike RBD in dilution buffer (0.3 mg/ml) and incubated for 1 hour at 37 °C. These mixtures were then immediately transferred to wells pre-coated with human ACE2 and incubated 1 hour at 37 °C. To remove any unbound salivary proteins the wells were washed thrice with wash buffer (from kit), and then developer solution (TMB substrate solution in kit) was incubated at 37 °C for 15 minutes before the stop solution was added. The optical density of each well was measured at 450 nm using a FLUOstar Optima microplate reader (BMG Labtech, Ottenberg, Germany). The intensity of assay signal decreased proportionally to the presence of neutralizing proteins.

### Virus propagation

An early-lineage SARS-CoV-2 isolate (hCoV-19/England/2/2020 obtained from PHE) was used in this study. For virus propagation, the African green monkey kidney cell line Vero E6 (provided by the Cell Services science technology platform (STP), The Francis Crick Institute, London, UK) was used. Vero E6 cells were grown in T75 flasks (156499, Radnor, PA, USA) in Dulbecco's Modified Eagle Medium (DMEM) supplemented with 5% FCS and 1× penicillin/streptomycin. Cells were fed three times a week and maintained at 37 °C and 5% CO_2_. These were passaged at 80%–90% confluency, detaching cells with trypsin-EDTA (VX25300-054, Gibco, Waltham, MA, USA). Vero E6 cells were infected with a multiplicity of infection (MOI) 0.01 pfu/cell in serum-free DMEM supplemented with 1% NEAA, 0.3% BSA, and 1X penicillin/streptomycin. The viruses were harvested after 7 days, then aliquoted and stored at −80 °C. The titer of virus was determined by plaque assay.

### Viral neutralization assay

Vero-E6 cells were seeded to a 96-well culture plate (Corning, flat-bottom wells, 3595), at 4 × 10^5^ cells per well, in 300 µL of media. These were incubated at 37 °C in 5% CO_2_ and grown until they reached confluency (approximately 24 hours). At this time, the SARS-CoV-2 virus stock was defrosted and diluted to the inoculum concentration of approximately 3000 pfu/mL in 2× minimal essential media (MEM), made from 10× MEM (Gibco, 21430020) in sterile water, with 4% FBS (Gibco, N4762), 1× L-glutamine (Thermo Fisher, 25030024), 1% penicillin/streptomycin (Thermo Fisher, 15140122), pH 7.35. 100 µL of saliva samples were then mixed with 100 µL of the viral inoculum (1:1 ratio) and incubated at 37 °C for 1 hour. The following control samples were also prepared in a 1:1 ratio and kept at 37 °C for 1 hour: saliva only (saliva sample and sterile 2× MEM), virus only (viral inoculum and 2× MEM), and negative control (2× MEM and sterile OptiMEM (Gibco, 11058021)). To initiate infection, 200 µL of culture media was removed from the Vero-E6 cells and replaced with 100 µL OptiMEM and 60 µL of sample or control per well, in triplicate, corresponding to approximately 100 CFU/well. Plates were then incubated at 37 °C in 5% CO_2_ for 48 hours. Media was discarded and the cells were fixed and stained with crystal violet (Sigma, V5265) in 20% ethanol, RT for 15 minutes. Wells were washed 2× with tap water to remove excess stain and air-dried. Cell loss was quantified by measuring the absorbance of the well at 590 nm using spiral well-scanning on the FLUOstar Omega (BMG Labtech, Ottenberg, Germany).

For analysis of target protein effect on VeroE6 infectivity, the assay was performed as above with the modification of replacement of whole saliva samples with dilutions in PBS of recombinant proteins vimentin (Cambridge Bioscience, Cambridge, UK), S100A9 and SERPINC1 (Fisher Scientific).

### Spike-baited mass spectrometry

The bait assay was adapted for saliva from a previously published method^44^. 10 µL of recombinant SARS-CoV-2 spike protein (50 µg/ml in PBS) was coated to each well of a deep 96-well plate using carbonate/bicarbonate buffer (100 mM at pH 9.6). The plate was left in the fridge overnight followed by washing with PBS. 200 µL of Horse myoglobin solution in PBS (1 mg/ml) was added to each well, incubated for 1 hour at RT, and then washed three times with PBS. 10 µL of 30% H_2_O_2_ was added to 75 µL aliquots of each saliva sample to quench any endogenous peroxidase activity in the saliva. 65 µL myoglobin buffer (0.05 mg/ml in PBS) was added to each sample. The aliquots of saliva samples were added to each well coated with spike or nucleocapsid. Plates were incubated for 1 hour at 37 °C after which time the supernatant was removed, plate washed three times with PBS-T and dried via centrifugation. 70 µL of Deoxycholate (DOC) (0.03 g/ml in 50 mM ammonium bicarbonate) was added to solubilize samples followed by 3 µL DTT for reduction and 6 µL IAA. 5 µL of Trypsin (5.6 mg/ml in 50 mM acetic acid) was added to each sample and incubated for 30 minutes at 45 °C for digestion. 5 µl of 6% Trifluoroacetic acid (TFA) was added to quench digestion. Samples were desalted and label-free proteomics analysis was performed as described previously^69^. Briefly, a 60 minute MS analysis was performed on an SYNAPT G2-Si mass spectrometer (Waters, Manchester, UK) in a Ultra Definition MSe (UDMSE) positive ion electrospray ionization mode. Raw MS data were processed using Progenesis QI analysis software (Nonlinear Dynamics, Newcastle-Gateshead, UK). Peptide identification was performed using MSe search identification against the UniProt Human reference proteome 2021, with one missed cleavage and a 1% peptide false discovery rate. Fixed modifications were set to carbamidomethylation of cysteines and dynamic modifications of oxidation of methionine peptides eluted as described previously before drying and reconstitution in acetonitrile and TFA. Samples were analyzed using a quadrupole-time of flight (Q-TOF) mass spectrometer.

### Immunofluorescence confocal microscopy of differentiated human nasal epithelial cell culture

The preparation and infection of air-liquid interface human nasal epithelial cell cultures for immunofluorescence staining were performed as previously described^70^. Images were captured using a LSM710 Zeiss (Zeiss, Oberkochen, Germany) confocal microscope and images were rendered and analyzed using Fiji/ImageJ v2.1.0/153c^71^.

### Statistical analysis

Data were initially collated using Microsoft Excel and statistical analysis was performed using GraphPad Prism version 9, SIMCA 17.0 (Umetrics, Sweden), and R running on RStudio v.1.2.5^72^. ELISA data were analyzed for individual comparisons using one-way analysis of variance with Tukey’s adjustment for multiple comparisons. For mass spectrometry, raw data was processed using ProteinLynx Global Server and Progenesis QI which identify proteins by searching the UniProtKB/Swiss-Prot database. Data was exported to Microsoft Excel for statistical analysis. The raw abundance data for each protein was normalized to the levels of spike or nucleocapsid to generate normalized abundance values. The Shapiro-Wilk test was used to test for normality. Fold changes were calculated based on the difference in mean protein levels between groups. Student’s t test was used to examine the differences in the mean protein levels between groups and SIMCA 17.0 for multivariable analysis. A *p* value < 0.05 was considered significant throughout.

## AUTHOR CONTRIBUTIONS

SE designed the study, conducted experiments, analyzed data, and prepared the manuscript. RW, AB, ID JKR, MW, TM, KMC, AC conducted experiments, analyzed data, and reviewed the manuscript. LEM, AC provided reagents, and experimental advice, and review of the manuscript. LR, IL, and AP assisted with data collection and review of the manuscript. TM supported work at BSL3. MW assisted with microscopy acquisition and reviewed the manuscript. ATO and MC-B conducted statistical analysis and reviewed the manuscript. WEH and KM contributed to mass spectrometry analysis and reviewed the manuscript. LG, SM, KCG, REH, and CMS oversaw the funding application and contributed to study conception and design, data analysis and interpretation, and the write-up of the manuscript.

## DECLARATION OF COMPETING INTEREST

All authors declare no conflicts of interest.

## FUNDING

This work was funded by GOSH Children’s charity (COVID_CSmith_017) and the NIHR Great Ormond Street Hospital Biomedical Research Centre. CMS was also supported by grants from Animal Free Research UK (AFR19-20274), BBSRC (BB/V006738/1) and the Wellcome Trust (212516/Z/18/Z). R.E.H. was supported by a Wellcome Trust Sir Henry Wellcome Fellowship (WT209199/Z/17/Z). Mass spectrometry was performed at the GOS ICH Mass Spectrometry Core Facility (UCL GOS Institute of Child Health, University College London, UK), which is supported by the NIHR GOSH BRC award 17DD08. Live virus experiments were performed in Containment Level 3 laboratory facilities at the UCL Centre for Clinical Microbiology at the Royal Free Hospital.
