# Supplementary material for: Salivary IgA and vimentin differentiate in vitro SARS-CoV-2 infection: A study of 290 convalescent COVID-19 patients
Source: Mucosal Immunol. 2024 Feb;17(1):124–36. doi: 10.1016/j.mucimm.2023.11.007 (PMC11139657; doi:10.1016/j.mucimm.2023.11.007)
Supplement: Supplementary data 1 [file mmc1.pdf]

Supplementary Figure 1

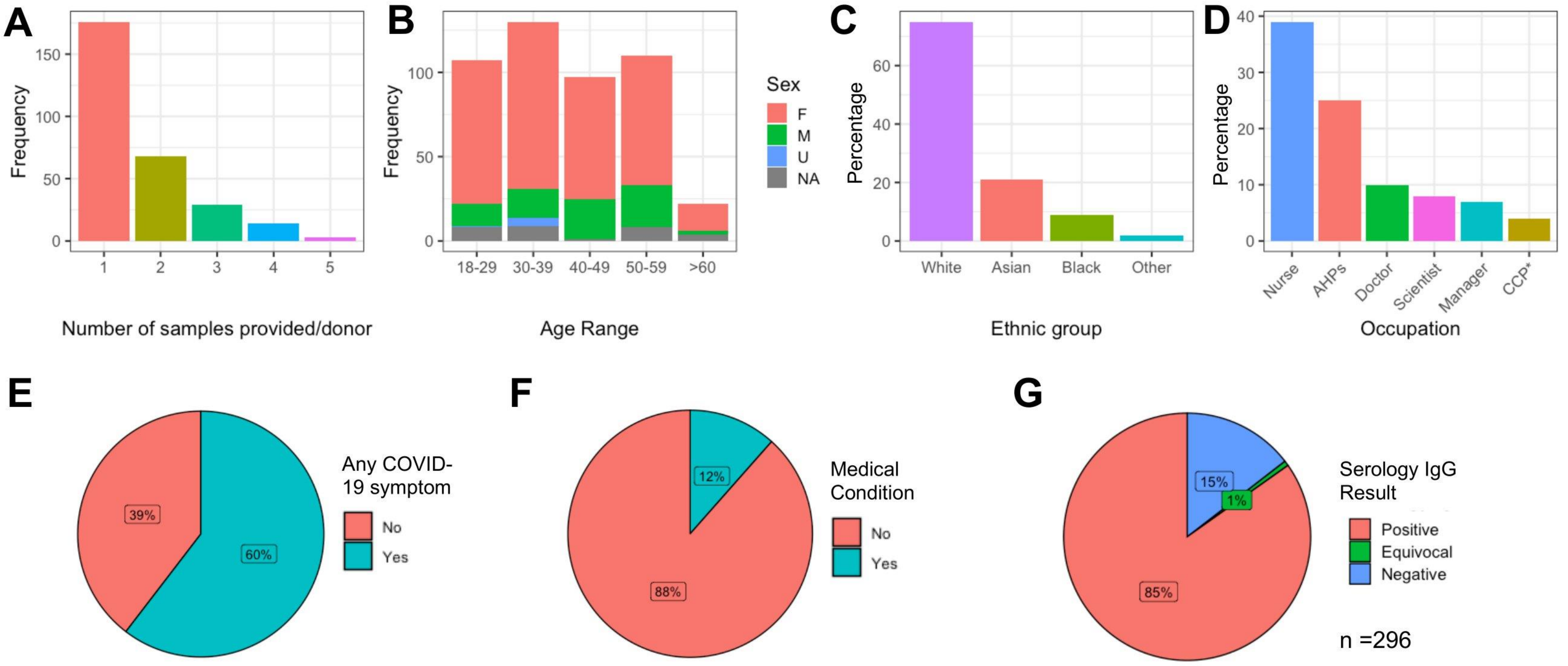

Supplementary Figure 1. Demographics of study population. A) Number of longitudinal saliva samples provided by study participants (n = 290). **B)** Number of study participants in different age groups and gender (n=270), including gender undeclared (U) and not available (NA). **C)** Number of study participants within different ethnic groups (n= 290). Here, over 75% of participants are classified as white. **D)** Number of participants within each NHS occupational category. Almost 70% of workers in this study were nurses or allied health professionals (AHPs) (n=242) *CCP = Cleaning, catering, or porters. **E)** Percentage of participants who reported having symptoms of COVID-19 prior to enrolment at positive PCR and/or serological test (n=290). **F)** Percentage of participants with underlying health issues outside the exclusion criteria (n=290). **G)** Percentage of participants who tested positive for anti-SARS-CoV-2 IgG in serology arm of Co-Stars study (n=296).

Supplementary Figure 2

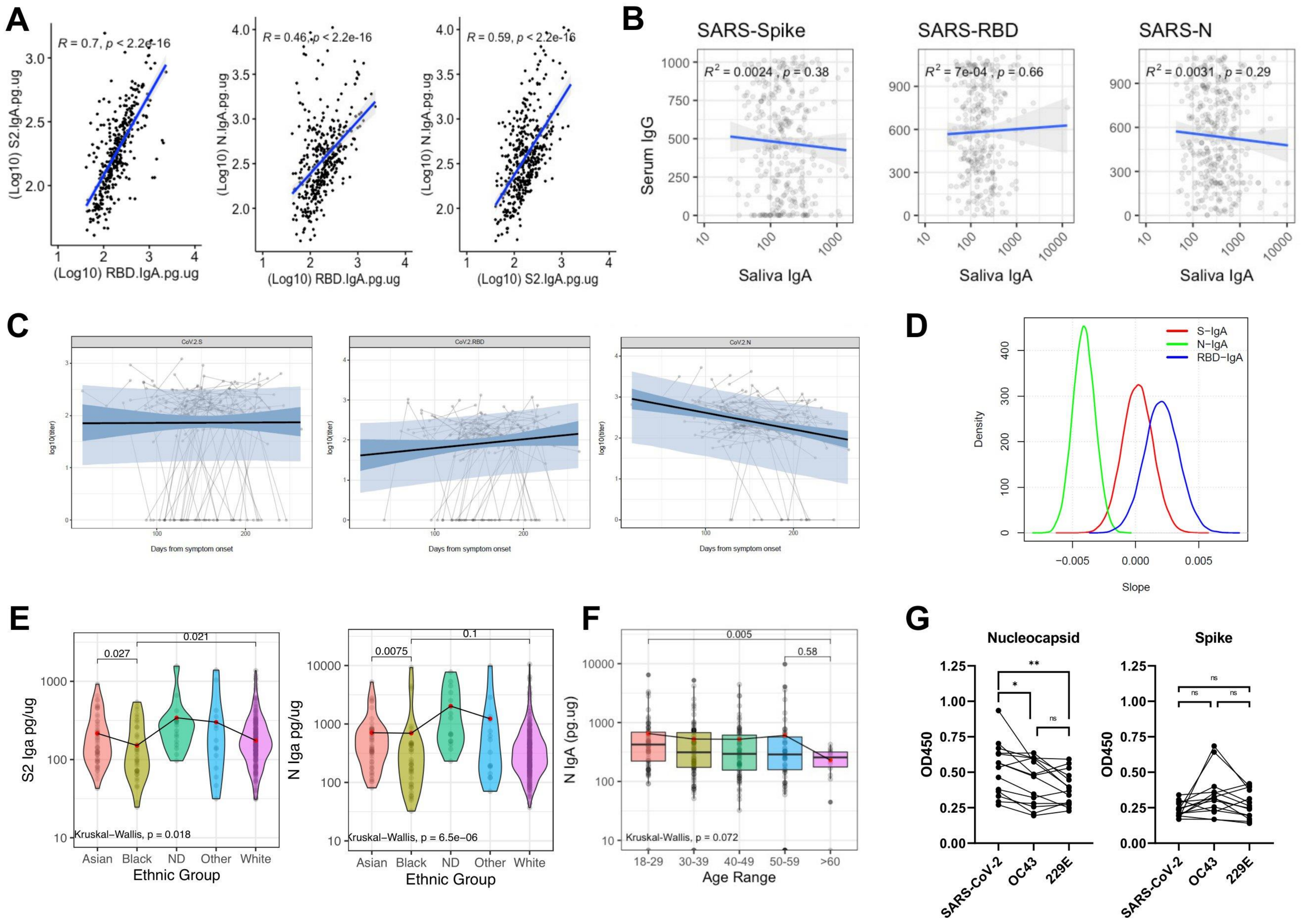

Supplementary Figure 2. Analysis of antibody correlations and persistence in convalescent health care workers A) A linear model fit was used to show the relationship between anti-S, anti-RBD and anti-N IgA concentrations, normalised to total protein (pg/µg). The value of r= denotes the value of the Pearson's coefficient and p-value of significance is given. (n=488) **B)** A linear model fit was used to show the relationship between saliva IgA and serology IgG for each SARS-CoV-2 antigen. **C)** Longitudinal IgA data from participants who provided repeated samples over time, which was used to calculate IgA decay rate using a constant exponential decay model. **D)** The IgA decay rate (slope represents change of log10 IgA titer per day) for each antigen as predicted by a constant exponential decay model. **E)** Anti-S and anti-N IgA by participant ethnicity, with red dots representing the mean for each group. **F)** Anti-N IgA was analysed by donor age group. Solid black bars denote the median and the red dot represents the mean for each age cohort. Kruskal-Wallis test was used to calculate significance overall and for specific groups using R. **G)** Salivary IgA recognition of N and S antigen from SARS-CoV-2 and seasonal coronaviruses OC43 and 229E (n=14).

Supplementary Figure 3

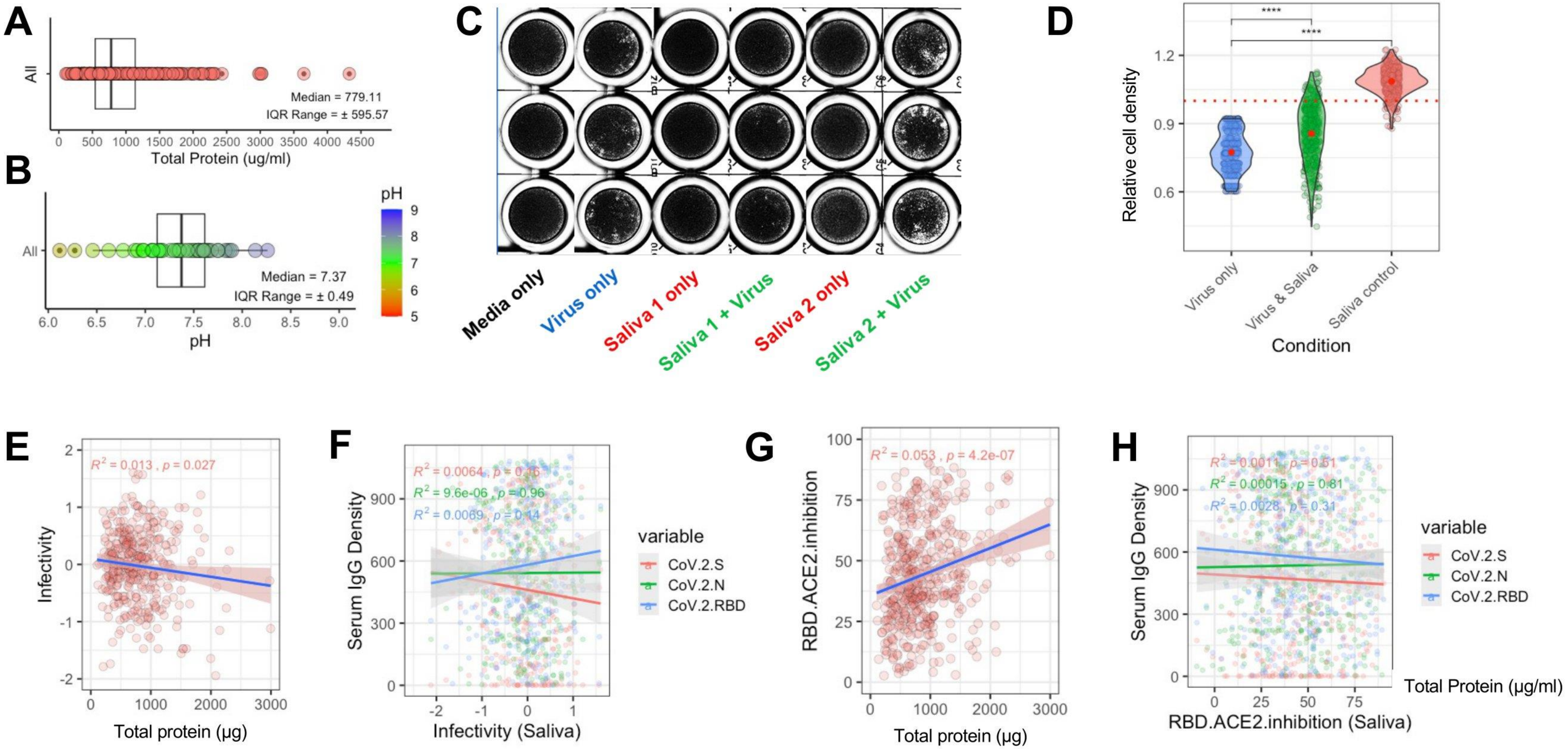

Supplementary Figure 3. Functional analysis of saliva from convalescent health care workers. A) The total protein concentration of all saliva samples (n = 488). **B)** The pH of a subset of the collected saliva samples (n=48). **C)** Assay design and representative images of the in vitro SARS-CoV-2 infectivity assay using VeroE6 epithelial cells stained with crystal violet. Sample 1 demonstrates reduction of relative infection and Sample 2 is representative of enhancement of infectivity. **D)** The endpoint VeroE6 cell density, expressed as a ratio versus untreated negative control (red line at ratio 1.0). Red dot denotes the median for each treatment. Kruskal-Wallis test was used to calculate significance overall and for specific groups using R (n=395). **E)** A linear model fit was used to show the relationship between relative infection of VeroE6 cells (fold-change versus virus-only controls) and total protein concentrations (n=395). The value of r = denotes the value of the Pearson's coefficient and p-value of significance is given. **F)** A linear model fit was used to show the relationship between the effect of saliva sample on VeroE6 cell infectivity and the matched serum IgG levels against SARS-CoV-2 antigens. **G)** A linear model fit was used to show the relationship between inhibition of RBD-ACE2 binding (%) and total protein concentrations (n=488). **H)** A linear model fit was used to show the relationship between the inhibition of RBD-ACE2 binding (%) by saliva and the matched serum IgG levels against SARS-CoV-2 antigens.

Supplementary Figure 4

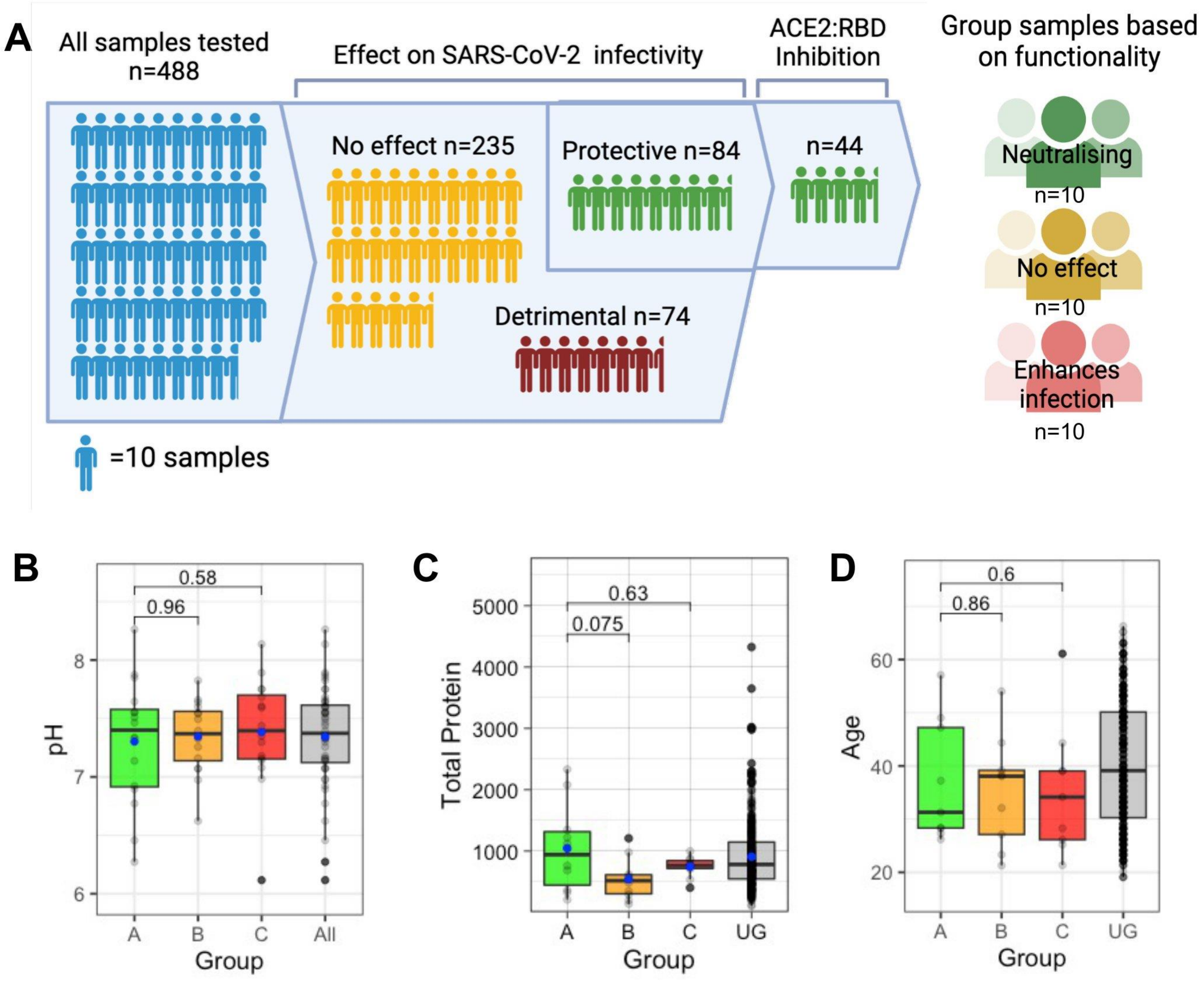

Supplementary Figure 4. Functional comparisons of saliva subsets selected for proteomics. A) A graphical representation of sample collection broken down by function. Protective effect on infectivity here represents FC>+0.5, detrimental effect represents FC<-0.5. ACE2-RBD inhibition represents >50% relative inhibition. **B)** Distribution of pH compared between subgroups A-C (n=10 each) and all pH screened samples (n=48). **C)** Distribution of total protein concentration (μg/ml) compared between subgroups A-C (n=10 each) and ungrouped (UG) samples (n=458). **D)** Distribution of donor age compared between subgroups A-C (n=10 each) and ungrouped (UG) samples (n=458). Black lines indicate median for each group, blue dot indicates mean value.

Supplementary Figure 5

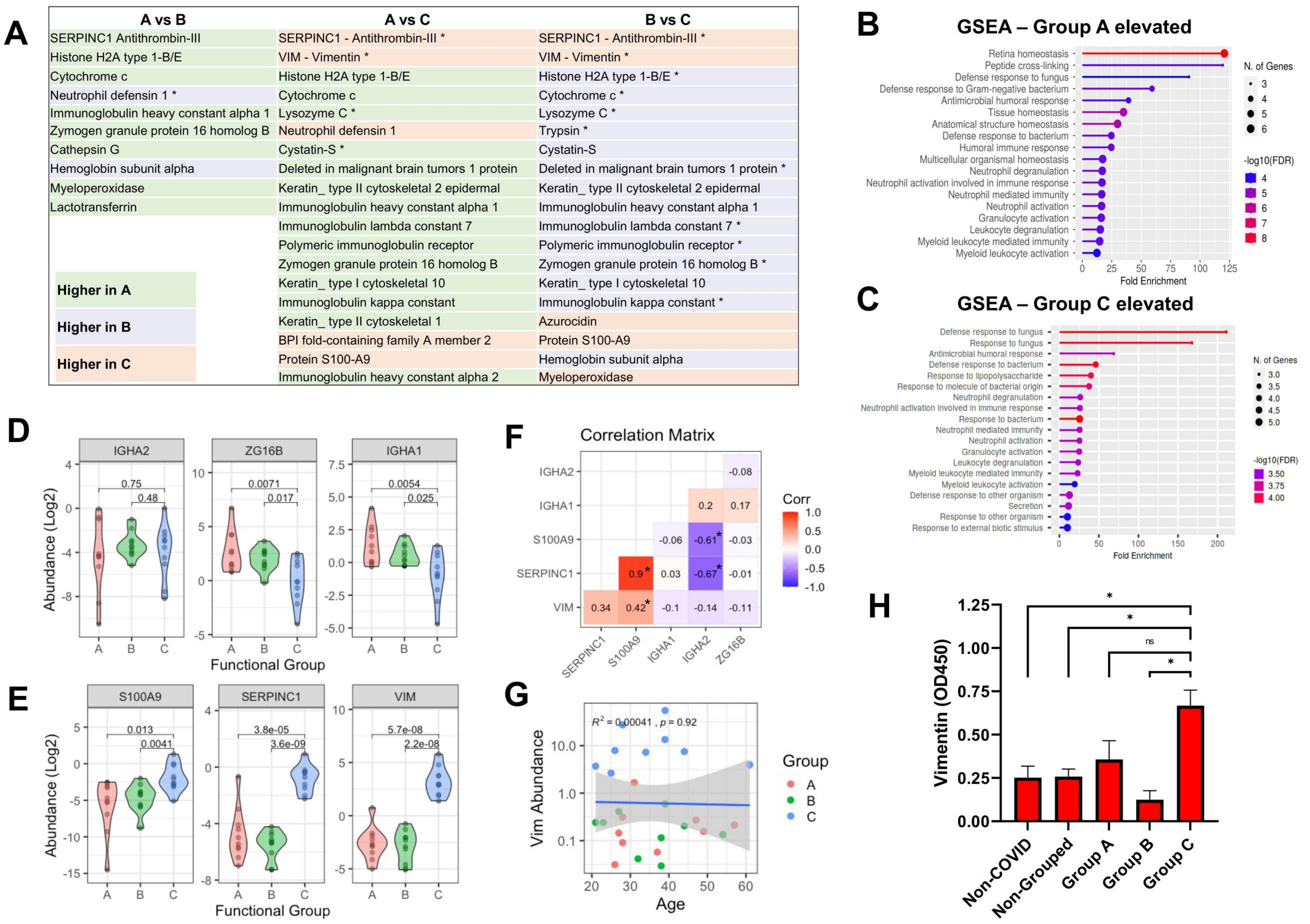

A vs B	A vs C	B vs C
SERPINC1 Antithrombin-III	SERPINC1 - Antithrombin-III *	SERPINC1 - Antithrombin-III *
Histone H2A type 1-B/E	VIM - Vimentin *	VIM - Vimentin *
Cytochrome c	Histone H2A type 1-B/E	Histone H2A type 1-B/E *
Neutrophil defensin 1 *	Cytochrome c	Cytochrome c *
Immunoglobulin heavy constant alpha 1	Lysozyme C *	Lysozyme C *
Zymogen granule protein 16 homolog B	Neutrophil defensin 1	Trypsin *
Cathepsin G	Cystatin-S *	Cystatin-S
Hemoglobin subunit alpha	Deleted in malignant brain tumors 1 protein	Deleted in malignant brain tumors 1 protein *
Myeloperoxidase	Keratin_ type II cytoskeletal 2 epidermal	Keratin_ type II cytoskeletal 2 epidermal
Lactotransferrin	Immunoglobulin heavy constant alpha 1	Immunoglobulin heavy constant alpha 1
	Immunoglobulin lambda constant 7	Immunoglobulin lambda constant 7 *
	Polymeric immunoglobulin receptor	Polymeric immunoglobulin receptor *
	Zymogen granule protein 16 homolog B	Zymogen granule protein 16 homolog B *
Higher in A	Keratin_ type I cytoskeletal 10	Keratin_ type I cytoskeletal 10
	Immunoglobulin kappa constant	Immunoglobulin kappa constant *
Higher in B	Keratin_ type II cytoskeletal 1	Azurocidin
	BPI fold-containing family A member 2	Protein S100-A9
Higher in C	Protein S100-A9	Hemoglobin subunit alpha
	Immunoglobulin heavy constant alpha 2	Myeloperoxidase

Supplementary Figure 5. Protein analysis of saliva functional subgroups. A) A summary of spike-binding proteins significantly elevated in group A, B or C. Proteins may be significantly elevated in group A (green), group B (blue) or group C (orange). **B)** GSEA pathway analysis for the proteins most highly expressed in Group A. **C)** GSEA Pathway analysis for the proteins most highly expressed in Group C. **D)** Violin plot showing the abundance of most differentially expressed spike-binding proteins detected in Group A (neutralising). **E)** Violin plot showing the abundance of most differentially expressed spike-binding proteins detected in Group C (detrimental). **F)** Correlation matrix showing the associations of vimentin (VIM), S100 calcium binding protein A9 (S100A9), antithrombin III (SERPINC1), Immunoglobulin heavy constant alpha chains (IGHA1 and IGHA2) and Zymogen Granule Protein 16B (ZG16B), as determined using the cor function of ggcorrplot package using R version 2023.03.1+446. (n=30; * represents significant correlation p <0.05) **G)** A linear model fit was used to show the relationship between vimentin abundance and donor ages, with the functional groups indicated by colour (n=27). **H)** Mean vimentin protein abundance quantified by ELISA, comparing representative samples from subgroups A-C with non-grouped saliva samples and saliva samples from non-COVID volunteer donors (n=31).

Supplementary Tables

Supplementary Table 1: Demographics and self-reported symptoms of study population

Age Groups	Count n	Gender			BAME			Any symptom		Any COVID-19 symptom		Abnormal Smell		Abnormal Taste		Cough		Fever	
		F	M	ND	Y	N	ND	Y	N	Y	N	Y	N	Y	N	Y	N	Y	N
**18-29**	79 (27.2%)	59 (74.7%)	13 (16.5%)	7 (8.9%)	13 (16.5%)	64 (81.0%)	2 (2.5%)	69 (87.3%)	10 (12.7%)	54 (68.4%)	25 (31.6%)	37 (46.8%)	42 (53.2%)	36 (45.6%)	43 (54.4%)	35 (44.3%)	44 (55.7%)	31 (39.2%)	48 (60.8%)
**30-39**	89 (30.7%)	70 (78.7%)	11 (12.4%)	8 (9.0%)	26 (29.2%)	62 (69.7%)	1 (1.1%)	71 (79.8%)	18 (20.2%)	52 (58.4%)	37 (41.6%)	40 (44.9%)	49 (55.1%)	41 (46.1%)	48 (53.9%)	42 (47.2%)	47 (52.8%)	30 (33.7%)	59 (66.3%)
**40-49**	60 (20.7%)	44 (73.3%)	15 (25.0%)	1 (1.7%)	21 (35.0%)	39 (65.0%)	0 (0.0%)	46 (76.7%)	14 (23.3%)	33 (55.0%)	27 (45.0%)	23 (38.3%)	37 (61.7%)	23 (38.3%)	37 (61.7%)	28 (46.7%)	32 (53.3%)	19 (31.7%)	41 (68.3%)
**50-59**	49 (16.9%)	35 (71.4%)	10 (20.4%)	4 (8.2%)	13 (26.5%)	35 (71.4%)	1 (2.0%)	40 (81.6%)	9 (18.4%)	30 (61.2%)	19 (38.8%)	25 (51.0%)	24 (49.0%)	28 (57.1%)	21 (42.9%)	22 (44.9%)	27 (55.1%)	23 (46.9%)	26 (53.1%)
**60<**	13 (4.5%)	10 (76.9%)	2 (15.4%)	1 (7.7%)	2 (15.4%)	11 (84.6%)	0 (0.0%)	11 (84.6%)	2 (15.4%)	7 (53.8%)	6 (46.2%)	3 (23.1%)	10 (76.9%)	4 (30.8%)	9 (69.2%)	8 (61.5%)	5 (38.5%)	5 (38.5%)	8 (61.5%)
**Total**	290	218 (75.2%)	51 (17.6%)	21 (7.2%)	75 (25.9%)	211 (72.8%)	4 (1.4%)	237 (81.7%)	53 (18.3%)	176 (60.7%)	114 (39.3%)	128 (44.1%)	162 (55.9%)	132 (45.5%)	158 (54.5%)	135 (46.6%)	155 (53.4%)	108 (37.2%)	182 (62.8%)

Supplementary Table 2: Longitudinal sample summary

Clinic visit number	Number of samples	Weeks post first sample (median± IQR)	Week interval (median± IQR)
1	290	-	-
2	113	5.6 (± 0.3)	5.6 (± 0.3)
3	45	9.8 (± 0.4)	4.9 (± 0.3)
4	17	13.4 (± 0.5)	4.1 (± 0.2)
5	3	14.7 (± 0.3)	3.3 (± 0.3)

Supplementary Table 3: Demographics and functionality of grouped saliva samples

																				Serum IgG (MSD)					VeroE6 assay	
Group	Person ID	Collection Week	Sex	Age	Age group	Abnormal Smell	Abnormal Taste	Hospital Admitted	Attended Hospital	Cough	Fever	Symptomatic	Asthma	BAME	Ethnic group	Height (cm)	Weight (kg)	BMI	Occupation	CoV.2.S	CoV.2.N	CoV.2.RBD	Total Protein (µg/ml)	RBD.ACE2 inhibition %	Saliva Toxicity	SARS-CoV-2 Infectivity (Log2 FC)
A	33958	28	F	57	50-59	Yes	Yes	No	No	Yes	Yes	Yes	No	Yes	Black	157	86	34.9	Porter	505	1074	546	2332.65	49.45	1.09	-6.64
A	-	30	-	-	-	-	-	-	-	-	-	-	-	-	-	-	-	-	-	-	-	-	1120.71	46.48	1.10	-1.47
A	30655	30	F	28	18-29	No	No	No	No	Yes	No	Yes	No	No	White	175	71	23.2	Nurse	542	575	614	1222.44	32.66	1.13	-4.32
A	32645	30	F	47	40-49	No	No	No	No	No	No	No	No	No	White	163	80	30.1	-	643	480	547	2079.38	23.28	1.08	-1.94
A	29047	31	M	37	30-39	No	No	No	No	No	No	No	No	Yes	Asian	171	91.9	31.6	Manager	1	222	312	1347.14	62.46	1.04	-2.12
A	31229	34	F	28	18-29	No	No	No	No	No	No	No	No	No	White	No	No	-	Nurse	25	371	186	208.46	68.67	1.07	-1.79
A	30987	34	F	49	50-59	No	No	No	No	Yes	No	Yes	No	No	White	No	No	-	Nurse	682	833	1008	326.59	22.96	1.17	-1.74
A	35784	34	F	31	30-39	Yes	No	No	No	Yes	No	Yes	No	No	White	No	No	-	AHPs	699	491	353	365.01	73.18	1.10	-1.36
A	31083	34	F	27	18-29	Yes	Yes	No	No	No	No	Yes	No	Yes	Black	No	No	-	-	48	275	132	762.08	29.38	1.10	-1.29
A	32771	40	-	26	18-29	Yes	Yes	No	No	No	Yes	Yes	No	No	White	No	No	-	Nurse	-	-	-	686.77	31.48	0.93	-4.06
B	-	30	-	-	-	-	-	-	-	-	-	-	-	-	-	-	-	-	-	-	-	-	1208.37	54.89	1.10	0.03
B	31307	30	F	39	40-49	No	No	No	No	Yes	Yes	Yes	No	Yes	Other	No	No	-	Nurse	1	828	483	346.78	41.71	1.08	0.01
B	29591	31	F	32	30-39	Yes	Yes	No	No	Yes	Yes	Yes	No	No	White	158	57	22.8	Doctor	656	521	226	289.3	15.93	1.18	0.03
B	38674	34	F	38	30-39	Yes	Yes	No	No	Yes	No	Yes	No	No	White	No	No	-	Nurse	953	400	898	981.25	24.03	1.10	0.03
B	31288	34	F	21	18-29	Yes	Yes	No	No	Yes	No	Yes	No	No	White	No	No	-	Nurse	297	168	237	138.73	9.41	1.15	0.01
B	37317	36	F	23	18-29	No	No	No	No	No	No	Yes	No	Yes	Black	No	No	-	Nurse	975	273	4	621.69	20.45	1.05	0.06
B	31173	37	F	54	50-59	Yes	Yes	No	No	No	Yes	Yes	No	No	White	No	No	-	AHPs	413	353	852	484.91	23.32	1.06	0.06
B	30280	38	F	44	40-49	No	No	No	No	No	No	No	No	No	White	166	58	21.0	Nurse	100	422	297	548.97	15.97	1.08	0.06
B	32693	38	F	27	18-29	Yes	Yes	No	No	No	No	Yes	No	No	White	No	No	-	Nurse	246	462	213	586.88	50.91	1.02	0.00
B	29199	43	F	38	30-39	Yes	Yes	No	Yes	Yes	No	Yes	No	Yes	Asian	163	70	26.3	-	-	-	-	195.06	20.47	1.15	0.07
C	29942*	28	F	39	40-49	No	No	No	No	Yes	Yes	Yes	No	No	White	164	72	26.8	Manager	319	398	325	997.71	42.57	1.08	1.05
C	31339	29	M	28	18-29	No	No	No	No	No	No	No	No	No	White	No	No	-	Doctor	1	469	278	780.64	19.90	1.06	1.02
C	29895	29	M	44	40-49	Yes	Yes	No	No	Yes	No	Yes	No	No	White	185	82	24.0	Doctor	557	849	866	707.72	27.41	1.10	1.60
C	-	30	-	-	-	-	-	-	-	-	-	-	-	-	-	-	-	-	-	-	-	-	747.72	41.63	1.13	1.49
C	29576	30	F	34	30-39	Yes	Yes	No	No	Yes	No	Yes	No	No	White	163	64	24.1	Nurse	471	585	234	790.88	52.87	1.14	1.58
C	30640	32	F	26	18-29	Yes	Yes	No	No	Yes	No	Yes	No	No	White	166	62	22.5	Nurse	287	868	977	878.59	50.00	1.11	0.95
C	29942*	32	F	39	40-49	No	No	No	No	Yes	Yes	Yes	No	No	White	164	72	26.8	Manager	319	398	325	744.72	81.67	1.03	1.03
C	30732	32	F	61	>60	No	No	No	No	No	No	Yes	No	No	White	No	No	-	AHPs	393	840	301	537.83	56.96	1.16	1.02
C	32015	41	F	21	18-29	No	No	No	No	No	No	No	Yes	No	White	171	81	27.7	-	nd	nd	nd	861.36	35.48	1.18	0.96
C	30338	44	F	25	18-29	No	No	No	No	No	No	Yes	No	No	White	No	No	-	Nurse	nd	nd	nd	402.1	34.59	1.12	0.93

- = not reported; nd = not detected

Supplementary Table 4: List of spike-binding salivary proteins identified by mass spectrometry

Protein name	Gene name	Abundance/Group		
		A	B	C
Albumin	ALB	1.82	3.60	2.13
Amylase 1B	AMY1B	31.91	37.05	37.42
Azurocidin	AZU1	0.07	0.05	0.10
BPI fold-containing family A member 2	BPIFA2	0.13	0.18	0.32
BPI Fold Containing Family B Member 2	BPIFB2	0.31	0.21	0.41
Carbonic Anhydrase 6	CA6	0.09	0.06	0.05
Cystatin SN	CST1	0.95	0.66	1.26
Cystatin C	CST3	0.00	0.00	0.00
Cystatin S	CST4	0.03	0.03	0.00
Cathepsin G	CTSG	0.12	0.04	0.07
Cytochrome C	CYCS	0.58	0.15	0.00
Neutrophil defensin 1	DEFA1	0.49	1.46	2.23
Deleted in malignant brain tumors 1 protein	DMBT1	0.38	0.36	0.02
Ecm29 Proteasome Adaptor And Scaffold	ECPAS	0.00	0.01	0.00
Histone H2A type 1-B/E	H2AC4	0.21	0.04	0.00
Hemoglobin subunit alpha	HBA1	0.02	0.06	0.02
Hemoglobin subunit beta	HBB	0.25	0.47	0.30
Immunoglobulin heavy constant alpha 1	IGHA1	4.17	1.71	0.83
Immunoglobulin heavy constant alpha 2	IGHA2	0.23	0.14	0.08
Immunoglobulin kappa constant	IGKC	0.26	0.26	0.10
Immunoglobulin lambda constant 7	IGLC7	0.17	0.18	0.05
Keratin_ type II cytoskeletal 1	KRT1	1.26	0.77	0.57
Keratin_ type I cytoskeletal 10	KRT10	1.17	0.97	0.26
Keratin_ type II cytoskeletal 2 epidermal	KRT2	0.06	0.08	0.01
Lactoperoxidase	LPO	0.67	0.89	0.66
Lactotransferrin	LTF	1.78	0.53	1.42
Lysozyme C	LYZ	0.20	0.10	0.00
Myoglobin	MB	27.12	37.65	45.44
Myeloperoxidase	MPO	0.55	0.17	0.51
Polymeric immunoglobulin receptor	PIGR	0.41	0.39	0.17
Prolactin Induced Protein	PIP	0.96	0.91	0.89
Serine Protease 1	PRSS1	0.12	0.14	0.00
Protein S100-A9	S100A9	0.07	0.07	0.44
Anti-thrombin III	SERPINC1	0.10	0.69	0.69
Vimentin	VIM	0.31	0.20	14.26
Zymogen granule protein 16 homolog B	ZG16B	16.58	4.91	1.95
